# Determinants of pancreatic tropism in metastatic renal cell carcinoma

**DOI:** 10.1172/jci.insight.195195

**Published:** 2026-03-09

**Authors:** Haitao Xu, Payal Kapur, Alana Christie, Aleksandra W. Nielsen, Averi Perny, Olivia Brandenburg, Charlotte Small, Jeffrey Miyata, Hua Zhong, Courtney Roberts, Roy Elias, Vanina Tcheuyap, Cassandra Duarte, Adrie van Bokhoven, Justine Panian, Haoran Li, Katharine A Collier, Debra Zynger, Luis Meza, Benoit Beuselinck, Neeraj Agarwal, Amir Mortazavi, Sumanta Pal, Rana McKay, Elaine T. Lam, Satwik Rajaram, James Brugarolas

**Affiliations:** 1 Department of Internal Medicine (Division of Hematology and Oncology),; 2 Kidney Cancer Program, Simmons Comprehensive Cancer Center,; 3 Department of Pathology,; 4 Department of Urology, and; 5 Department of Bioinformatics, University of Texas Southwestern Medical Center, Dallas, Texas, USA.; 6 Department of Medicine, University of Colorado Cancer Center, and; 7 Department of Pathology, University of Colorado Anschutz Medical Campus, Aurora, Colorado, USA.; 8 Department of Medicine, Moores Cancer Center, UCSD, San Diego, California, USA.; 9 Department of Internal Medicine, Huntsman Cancer Institute, University of Utah, Salt Lake City, Utah, USA.; 10 Department of Internal Medicine, The Ohio State University Comprehensive Cancer Center, Columbus, Ohio, USA.; 11 Department of Pathology, The Ohio State University, Columbus, Ohio, USA.; 12 The Department of Medical Oncology & Therapeutics Research, City of Hope, Duarte, California, USA.; 13 Department of General Medical Oncology, University Hospitals Leuven, KU Leuven, Leuven, Belgium.

**Keywords:** Clinical Research, Oncology, Cancer

## Abstract

**BACKGROUND:**

Clear cell renal cell carcinoma (ccRCC) with pancreatic metastases (PM) is paradoxically associated with prolonged overall survival (OS), but the biological basis for this observation remains unclear.

**METHODS:**

We analyzed matched primary and metastatic samples from an international consortium of patients with PM (*n* = 108) and compared them with a previously characterized ccRCC cohort without PM (*n* = 273).

**RESULTS:**

Primary ccRCC tumors associated with PM were dominated by indolent, angiogenic phenotypes, characterized by low-grade histology and reduced mTORC1 activation (all *P* < 0.001). Tumors of patients with PM were often PBRM1-deficient (80.4% vs. 54.8%, *P* < 0.001) and rarely harbored BAP1 loss (3.7% vs. 20.7%, *P* < 0.001). After metastasis diagnosis, patients with PM had significantly longer median OS compared with those without PM (110 vs. 33 months, HR 0.28 [95% CI, 0.19–0.39], *P* < 0.001). Survival was further prolonged among patients with PBRM1 loss (143 vs. 64 months, HR 0.41 [95% CI, 0.22–0.81], *P* = 0.008). Notably, PM lesions were typically low-grade and PBRM1-deficient even when more aggressive and evolved clones were present in primary tumors. Finally, PBRM1 loss was associated with preferential response to angiogenesis inhibitors over immune-oncology therapy, reflected by longer time on treatment (32.1 vs. 9.1 months, HR 0.16 [95% CI, 0.06–0.39], *P* < 0.001).

**CONCLUSION:**

These findings illustrate selective tropism of indolent, less-evolved, PBRM1-deficient ccRCC clones for pancreatic dissemination. This biological bias likely underlies therapeutic sensitivity and favorable survival, supporting the consideration of PBRM1 status and metastatic tropism in risk stratification and treatment selection.

**FUNDING:**

NIH Kidney Cancer SPORE grant (P50CA196516); The Cancer Prevention and Research Institute of Texas (RP220294); Endowment from Jan and Bob Pickens Distinguished Professorship in Medical Science and Brock Fund for Medical Science Chair in Pathology.

## Abstract 2

Pancreatic metastasis arises predominantly from indolent clones within primary RCC with distinct molecular and histological features, clinical behavior, and treatment responsiveness.

## Introduction

Historically, metastatic renal cell carcinoma (mRCC) is associated with a 5-year survival rate of less than 20% (1), although more recent clinical trials have demonstrated a median overall survival (OS) of approximately 50 months (2, 3). However, mRCC is quite heterogeneous, and while some patients have metastases at presentation, other patients develop metastases after several years, including sometimes more than 5 years (1, 4, 5). Interestingly, both metastatic latency and tropism have been associated with improved OS. Specifically, metastases to endocrine organs correlates with better OS. In particular, patients with pancreatic metastases (PMs) fare significantly better (6–10). However, PMs are uncommon (11, 12).

Previously, we reported that RCCs that metastasize to the pancreas have infrequent loss of BAP1, a tumor suppressor protein that is mutated in approximately 15% of metastatic clear cell RCC (ccRCC) and is associated with a high grade and poor OS (13–15). Furthermore, tumors with PMs were enriched for PBRM1 loss, which is associated with a low grade and better OS (9). However, the association with PBRM1 loss was inconclusive, which we attributed to the small cohort size. In this study, we expand upon our prior work and further investigate the molecular determinants of PM tropism by leveraging samples from a multicenter, international consortium of patients with PM and detailed clinical outcomes (8). By integrating patient characteristics, histological tumor features, and genomic alterations with treatment response and outcomes, we provide insights into the unique biological nature of PM in RCC.

## Results

### Clinicopathological characteristics of patients with PMs.

This study focuses on a subset of patients with clear cell histology and samples available from an international, clinical PM consortium study (8). First, we sought to assess how the subset of patients in the current study compared with the original report. In the original study, patients were split into 2 groups and integrated data were not available (see Methods, *Study design and patient population*). Thus, to assess the representativeness of the cohort with samples, comparisons were performed to each of the groups. Notwithstanding the focus on ccRCC in the current study, these analyses showed that clinicopathological characteristics between the clinical and the sample cohorts were similar (Supplemental Tables 1 and 2; supplemental material available online with this article; https://doi.org/10.1172/jci.insight.195195DS1). Two items had a standardized difference greater than 0.3. First, there was a greater proportion of White patients in the subset with samples available in group 1. Second, the proportion of patients with local treatment for PMs was greater in the sample cohort (27.3% vs. 14.9%). Although there is not an obvious explanation for the former, the later was expected since the current study focuses on patients with available tumor samples (including PMs), which would have been retrieved through focal therapy (i.e., surgery).

Having established the representativeness of the current PM cohort (*n* = 108), we next assessed how these patients compared with patients with ccRCC without PM. For these studies, we retrieved a previously assembled cohort of patients with mRCC without PM (*n* = 273) (9). As a reference, the control cohort has a comparable median OS to patients with metastatic ccRCC in contemporaneous clinical trials, such as the METEOR and CheckMate 214 (16, 17). Compared with the non-PM reference cohort, patients with PM were diagnosed with RCC at earlier stages (stage 1 and 2, 33.3% vs. 14.1%, *P* < 0.001) and their primary tumors were more frequently of lower grade (42.7% vs. 22.1%, *P* < 0.001) (Table 1). In addition, patients with PM developed metastases significantly later than patients without PM (median [IQR]: 3 [0–7] years vs. 0 [0–1] years, *P* < 0.001), which is consistent with an association between PM and metastatic latency (4). In keeping with the late onset of metastases, the PM patient group was also more likely to be in a favorable risk category (49.6% vs. 21.2%, *P* < 0.001), as categorized by the International Metastatic Renal Cell Carcinoma Database Consortium (IMDC).

### Patients with PM have longer survival.

**Figure 1:**
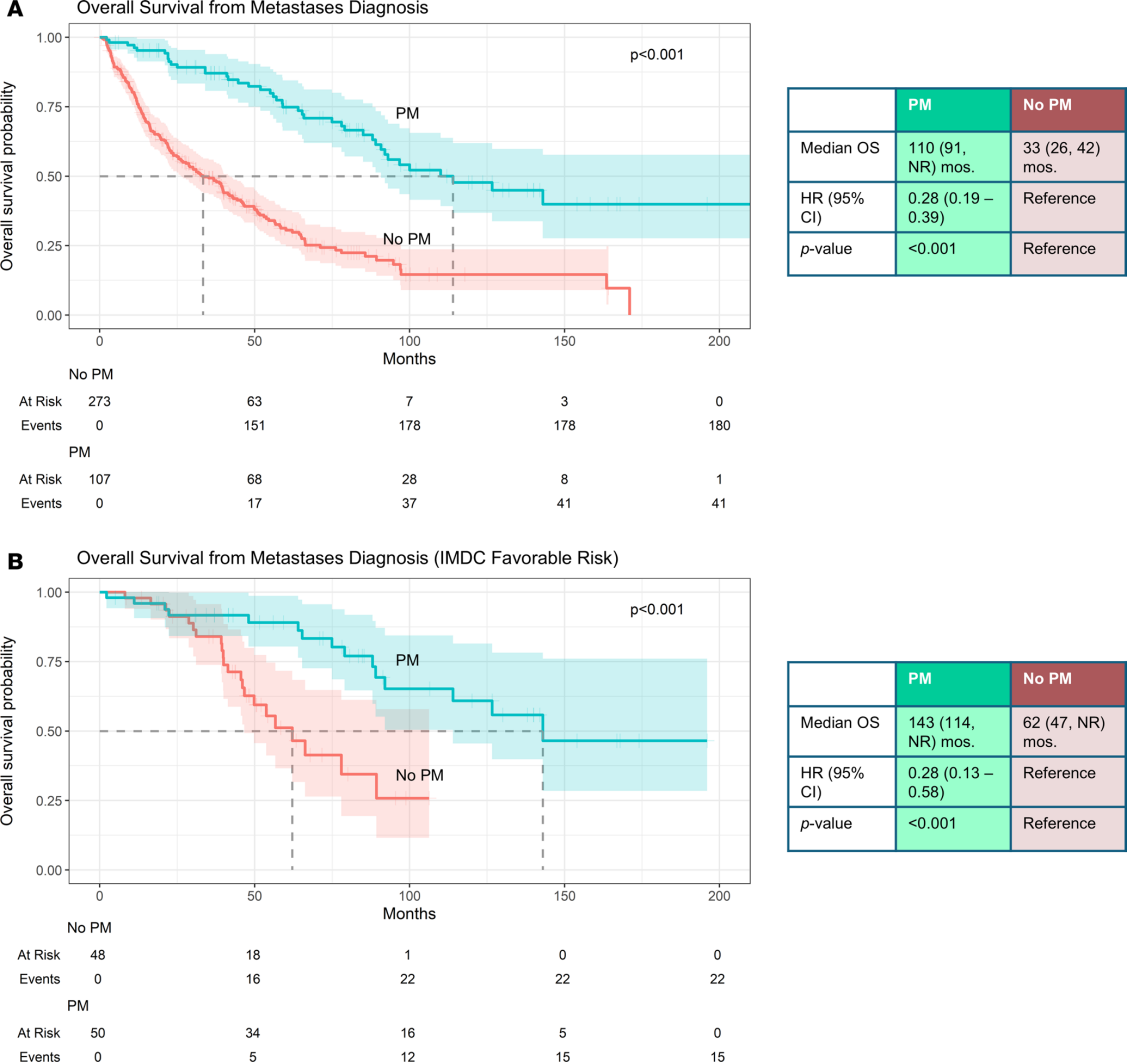
Kaplan-Meier analyses of overall survival for patients with mRCC with or without pancreatic metastasis. Kaplan-Meier and Cox proportional hazards analyses of OS from time of metastases diagnosis for (**A**) all patients and (**B**) those with favorable IMDC risk profile. Patients with missing survival data were excluded. OS, overall survival; PM, pancreatic metastasis; No PM, cohort without pancreatic metastases.

We compared OS between the PM and non-PM cohort. Even after adjusting for metastatic latency by evaluating OS from the time of metastasis diagnosis, patients with PM exhibited a significantly longer median OS compared with patients without PM (110 vs. 33 months, HR 0.28 [95% CI, 0.19–0.39], *P* < 0.001) (Figure 1A). Notably, the improvement in survival persisted even among patients in a favorable IMDC risk category (HR 0.28 [95% CI, 0.13–0.58], *P* < 0.001) (Figure 1B).

We next assessed the potential contribution of clinicopathological features to OS. Unstained slides from representative clinical blocks containing the highest-grade areas of the primary tumor were stained and subjected to central review (reviewer blinded to clinical data), and assigned a nucleolar grade. For patient-level comparisons, tumor grade was based on data reported in the standardized template provided by each participating institution. Although some differences were observed between the grade reported and the centralized review (Supplemental Figure 1), they were not statistically significant (see Methods).

A multivariable analysis controlling for race, sex, and other significant variables identified in univariate analyses showed that the presence of PM remained a strong and significant predictor of OS (Supplemental Figure 2).

### PBRM1 loss is associated with prolonged survival.

We and others have previously shown that PBRM1 loss is associated with prolonged survival, especially when compared with patients whose tumors are BAP1-deficient (13, 18, 19). We compared the frequency of BAP1 and PBRM1 loss across the 2 cohorts. To evaluate the status of these tumor suppressor proteins, we performed IHC studies. Interpretable results were obtained for 107 out of 108 patients with PM for both BAP1 and PBRM1, and we compared these results to previously available results from the control cohort (Table 2). We previously reported that BAP1 loss occurred infrequently in ccRCC with PM (9), and this was confirmed in the current study (3.7% vs. 20.7%; *P* < 0.001). In the current study, we also showed that PM was frequently associated with PBRM1 loss (80.4% vs. 54.8%; *P* < 0.001), a finding that did not reach statistical significance in our prior publication (9).

We evaluated clinicopathological characteristics of patients with PMs according to PBRM1 status. We compared the 86 patients with PBRM1-deficient tumors (80.4%) with the 21 patients with retained PBRM1 (19.6%) (Table 3). Among the different variables evaluated (demographics, stage at diagnosis, grade assigned by institutions, IMDC risk category, ECOG performance status, etc.) only ECOG performance status differed. Although the size of the cohorts compared was small, we found that patients with PBRM1-deficient tumors (PBRM1^–^) exhibited better ECOG performance status at diagnosis (*P* = 0.025).

**Figure 2:**
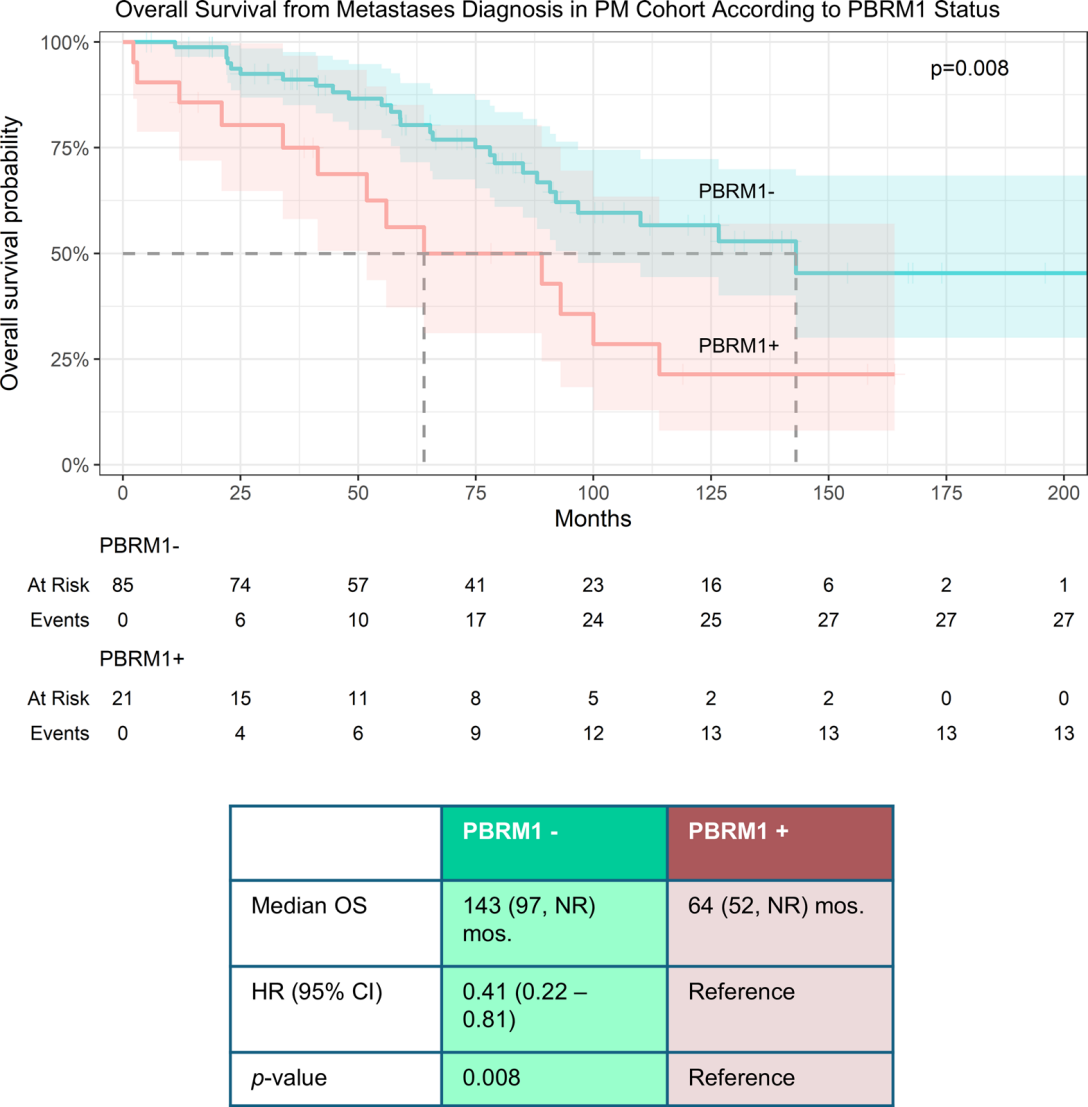
Kaplan-Meier analyses of patients with pancreatic metastasis according to PBRM1 status. Kaplan-Meier and Cox proportional hazards analyses of OS from time of metastases diagnosis for patients with PM according to PBRM1 status. OS, overall survival; PBRM1^–^, PBRM1-deficient tumor; PBRM1^+^, tumor with retained PBRM1; PM, pancreatic metastasis.

Next, we compared survival outcomes. Median OS from metastasis diagnosis was significantly longer in the PBRM1^–^ group compared with the PBRM1^+^ group (143 vs. 64 months, HR 0.41 [95% CI, 0.22–0.81], *P* = 0.008) (Figure 2). Notably, PBRM1 status remained an independent predictor of OS even after controlling for ECOG performance status (or IMDC risk profile) (Supplemental Figure 3). These data suggest that PBRM1 is prognostic in patients with PMs.

### Improved outcomes with angiogenesis inhibitors in patients with PMs and PBRM1 loss.

**Figure 3:**
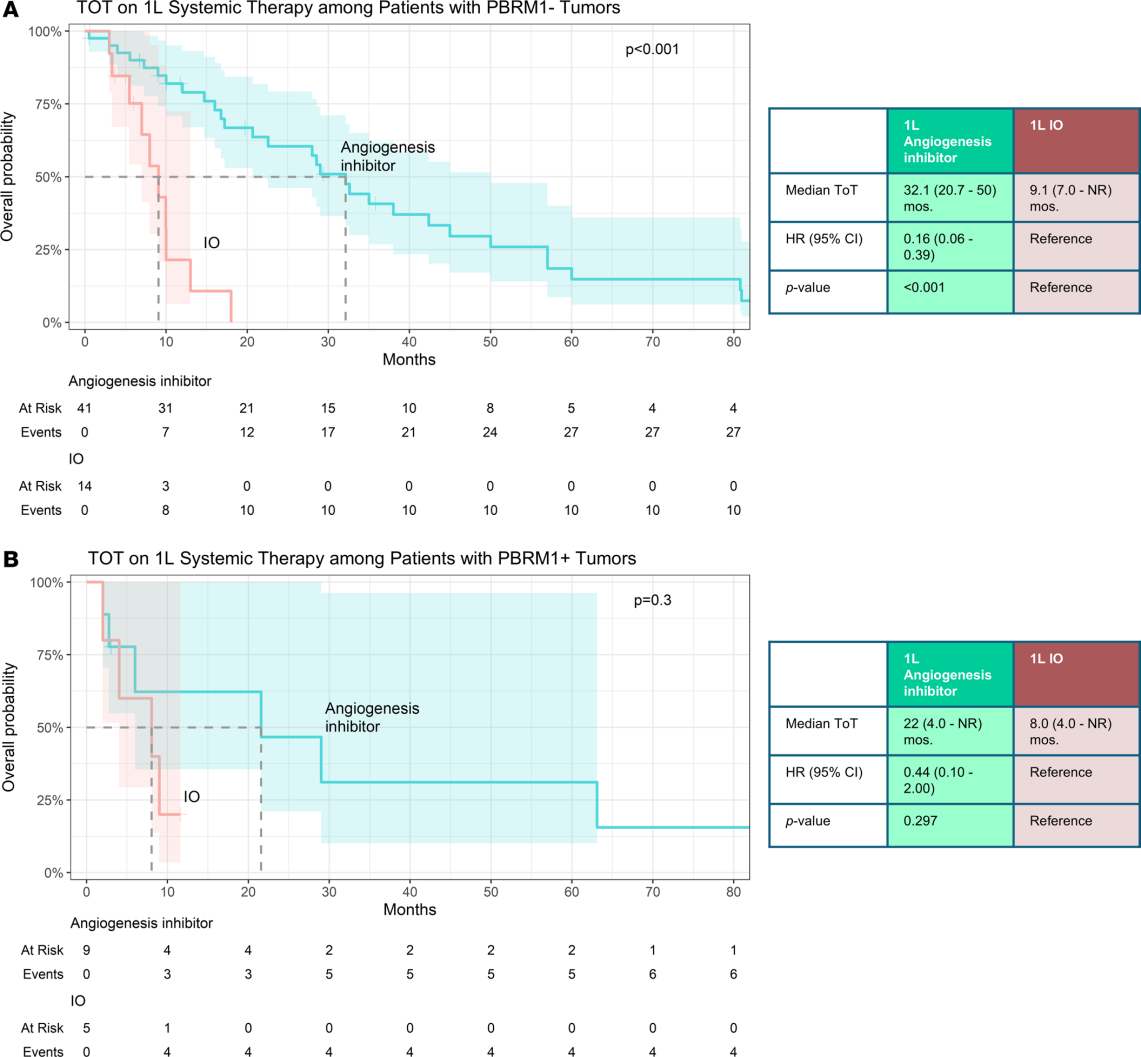
Kaplan-Meier analyses of patients with pancreatic metastasis according to treatment type. Kaplan-Meier and Cox proportional hazards analyses of TOT (angiogenesis inhibitor and IO therapy) for patients with PM with (**A**) PBRM1-deficient tumors and (**B**) PBRM1-WT. IO, immune-oncology therapy; PBRM1^–^, PBRM1-deficient tumor; PBRM1^+^, tumor with retained PBRM1; PM, pancreatic metastasis; TOT, time on treatment.

Previous studies have suggested an association between PBRM1 loss and response to angiogenesis inhibitors (20–22). Thus, we examined the impact of PBRM1 status on treatment response. Among 107 patients with PMs and available PBRM1 status, first-line (1L) therapy was as follows: 53 received an angiogenesis inhibitor, 19 an immune-oncology (IO) drug, and 7 a combination of angiogenesis inhibitor/IO drug. The remaining 28 patients did not receive systemic therapy. We then examined time on treatment (TOT), a surrogate for progression-free survival employed in the original consortium study (8). Patients with PBRM1-deficient tumors had significantly longer TOT with 1L antiangiogenic therapy (32.1 months, 95% CI, 20.7–50) compared with IO therapy (9.1 months, 95% CI, 7.0–NR; HR 0.16 [95% CI, 0.06–0.39], *P* < 0.001) (Figure 3A). In patients with PBRM1^+^ tumors, TOT differences between angiogenesis inhibitors (22 months, 95% CI, 4.0–NR) and IO therapy (8.0 months, 95% CI, 4.0–NR) were not statistically significant (HR 0.44 [95% CI, 0.10–2.00], *P* = 0.297), but these results are limited by small numbers, and the trends were similar in both groups (Figure 3B). Overall, the data suggest that patients with PM and PBRM1 loss preferentially respond to antiangiogenic therapy.

### Patients with both pancreatic and extrapancreatic metastases exhibit more indolent features compared with patients without PMs.

Next, we focused on the subset of patients with both pancreatic and extrapancreatic metastases. This subset accounted for 77 of 108 (71%) patients. Compared with the non-PM reference cohort, patients that had extrapancreatic metastases were also diagnosed with RCC at earlier stages (31.7% vs. 14.1%, *P* = 0.001), had primary tumors of lower grade (40.0% vs. 22.1%, *P* = 0.018), and were more likely to be in a favorable IMDC risk category (46.0% vs. 21.2%, *P* < 0.001) (Supplemental Table 3). In addition, patients with extrapancreatic metastases also developed metastases significantly later than patients without PM (median [IQR]: 2 [0–6] years vs. 0 [0–1] years, *P* < 0.001), reinforcing the association between tropism to the pancreas and metastatic latency (Supplemental Table 3). Even among patients with both pancreatic and extrapancreatic metastases, median OS remained significantly longer compared with patients without PM (127 vs. 33 months; HR 0.26 [95% CI, 0.17–0.39]; *P* < 0.001) (Supplemental Figure 4A). Additionally, the presence of PM continued to be a significant predictor of prolonged survival after adjusting for differences in clinicopathological characteristics (Supplemental Figure 4B). Even in this population, PBRM1 loss still independently predicted improved OS (NR vs. 64 months; HR 0.26 [95% CI, 0.18–0.88]; *P* = 0.022) (Supplemental Figure 5A). PBRM1 loss also correlated with prolonged TOT with angiogenesis inhibitors compared with immunotherapy in this group of patients (Supplemental Figure 5B).

### Primary tumors from patients with PM differ from those without PM.

Next, we performed more detailed analyses of primary tumors and metastases. We focused initially on primary tumors (*n* = 72), which were available for 71 patients (1 patient with tumors in both kidneys). For these experiments, we compared primary tumor samples from patients with PM to a cohort of 110 previously characterized primary tumor samples from patients with metastasis without PM (9, 23).

**Figure 4:**
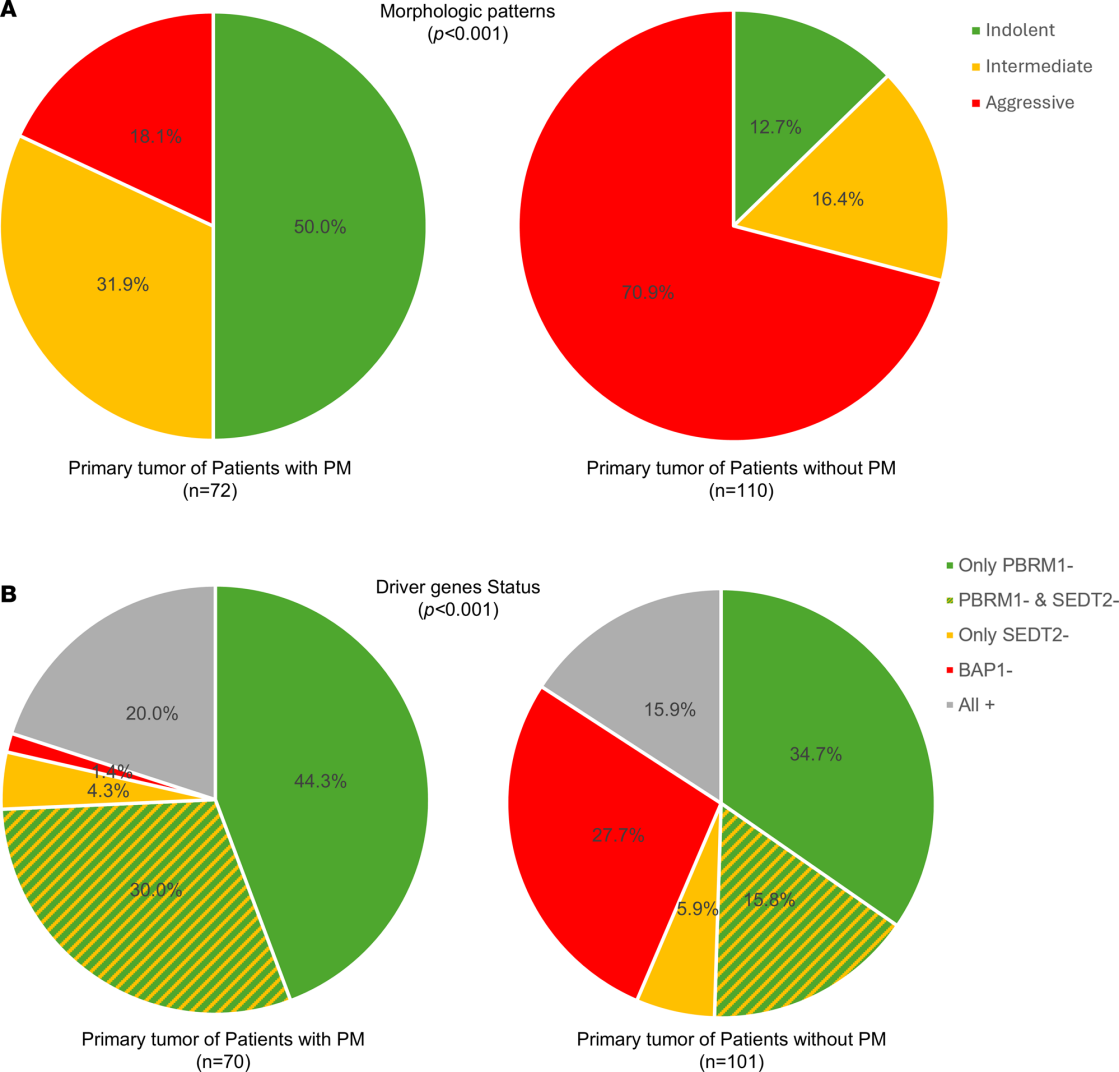
Primary tumors from patients with pancreatic metastasis differ from those without. Integrated pie charts of (**A**) dominant architectural patterns and (**B**) driver gene status (by IHC) of primary tumors from patients with or without PM, compared using the Fisher-Freeman-Halton exact test. BAP1/PBRM1/SETD2 status were examined together. Tumors with BAP1 loss, regardless of the status of other IHCs, were considered BAP1^–^. The remaining tumors were categorized as “Only PBRM1^–^,” “Only SETD2^–^,” or “PBRM1^–^ and SETD2^–^” based on PBRM1/SETD2 status. Any remaining tumors with retained BAP1, PBRM1, and SETD2 were considered as “All^+^.” Samples with missing values for PBRM1, BAP1, or SETD2 were excluded. PM, pancreatic metastasis.

First, we focused on tumor architecture. We found that primary tumors from patients with PM were significantly enriched in indolent architectural patterns (50.0% vs. 12.7%, *P* < 0.001) and had a lower frequency of aggressive architectures (18.1% vs. 70.9%, *P* < 0.001) (Figure 4A). Next, we evaluated how driver gene status compared between primary tumors in both cohorts. For this study, BAP1/PBRM1/SETD2 status were examined together, similar to previous studies (4). Tumors with only PBRM1 loss were categorized as “Only PBRM1^–^”; tumors with only SETD2 loss were categorized as “Only SETD2^–^”; and tumors with loss of both PBRM1 and SETD2 were categorized as “PBRM1^–^ and SETD2^–^.” Given that loss of BAP1 is associated with higher aggressiveness, any tumors with BAP1 loss, regardless of the status of the other genes, was considered “BAP1^–^.” The remaining tumors with retained BAP1, PBRM1, and SETD2 were considered as “All^+^.” We found that 74.3% of PM primary tumors had PBRM1 loss (with or without SETD2 loss, which cooperates with PBRM1 loss; refs. 15, 24), and PBRM1 loss was observed in 50.5% tumors without PM (*P* = 0.002). Conversely, BAP1 loss frequencies were significantly lower in primary tumors from patients with PM (1.4% vs. 27.7%, *P* < 0.001) (Figure 4B). Thus, primary tumors of patients with PM are characterized by indolent architectures and PBRM1 loss.

### PMs are characterized by indolent features.

**Figure 5:**
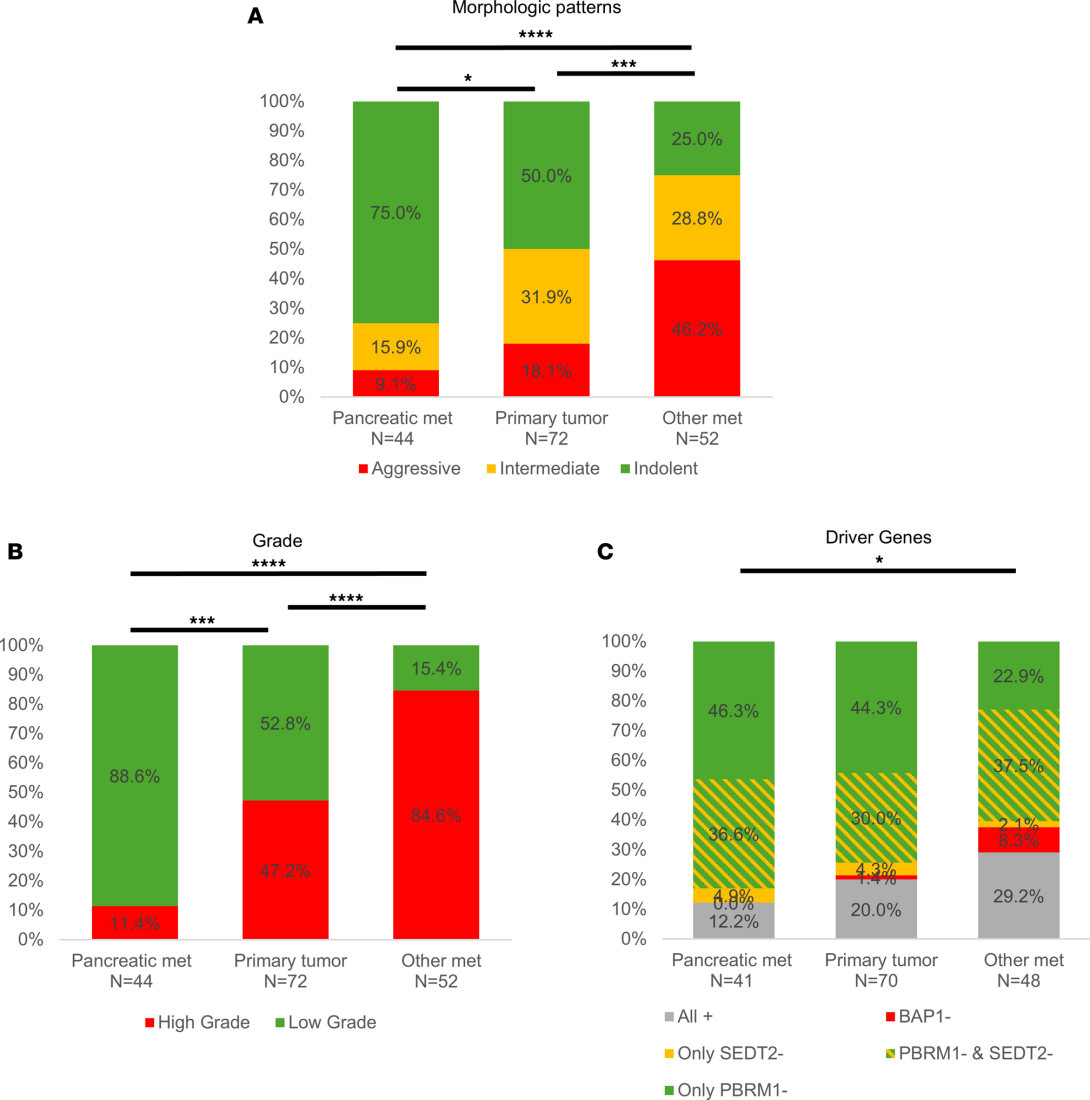
Pancreatic metastasis samples are characterized by indolent histology and molecular features. Integrated stacked figure of (**A**) dominant architectural patterns, (**B**) tumor grade determined by centralized review (see Methods), and (**C**) driver gene status in primary tumors and metastatic samples from patients with PM. For this study, BAP1/PBRM1/SETD2 status were examined together: Tumors with only PBRM1 loss were categorized as “Only PBRM1^–^”; tumors with only SETD2 loss were categorized as “Only SETD2^–^”; tumors with loss of both PBRM1 and SETD2 were categorized as “PBRM1^–^ and SETD2^–^”; any tumors with BAP1 loss, regardless of the status of the other genes, were categorized as “BAP1^–^”; remaining tumors with retained BAP1, PBRM1, and SETD2 were categorized as “All^+^.” Samples with missing values for PBRM1, BAP1, or SETD2 were excluded. *P < 0.05, ***P < 0.001, and ****P < 0.0001 by Fisher’s exact test or the Fisher-Freeman-Halton exact test, as appropriate.

Next, we focused our analyses on how metastases related to the corresponding primary tumors in patients in the PM cohort. For tissue-level comparisons, tumor grade was based on the data assigned after centralized review by an expert pathologist (see Methods). We investigated both PM (44 PM samples; 44 patients) as well as metastases at other sites (53 nonpancreatic “other” metastases; 34 patients). Notably, compared with primary tumor samples, PM samples were more indolent (75.0% vs. 50.0%, *P* = 0.021) and of lower grade (88.6% vs. 52.8%, *P* < 0.001) (Figure 5). In contrast, non-PM metastases had more aggressive patterns compared with primary tumors (46.2% vs. 18.1%, *P* < 0.001) and higher grade (84.6% vs. 47.2%, *P* < 0.001) (Figure 5, A and B). Thus, while metastases to sites other than the pancreas were characterized by aggressiveness, PMs were not. Furthermore, the higher frequency of indolent architectures and lower grade in PMs compared with primary tumors suggest that the pancreas is preferentially seeded by more indolent clones. These morphological differences between PMs and other metastases correlated with driver genes (*P* = 0.023). Whereas PMs were enriched for PBRM1 loss (alone or in combination with SETD2 loss), rates were lower in other metastases (82.9% vs. 60.4%, *P* = 0.034; Figure 5C).

**Figure 6:**
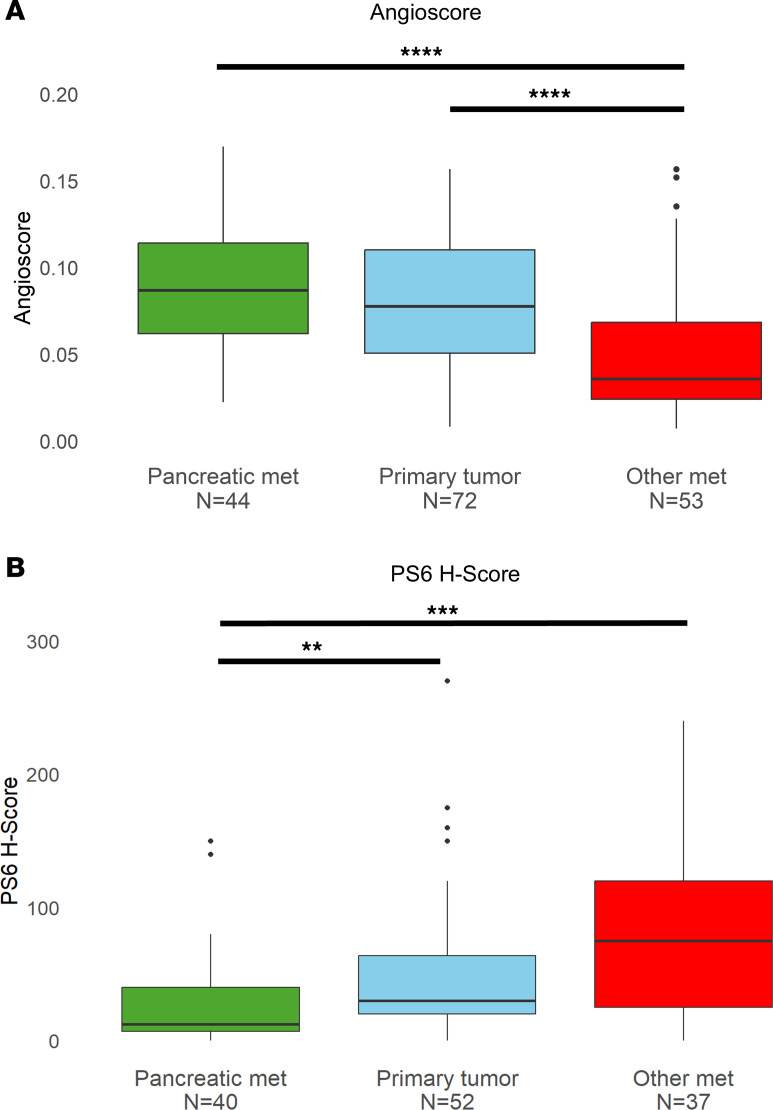
Pancreatic metastasis samples are characterized by heightened angiogenesis and lower mTOR pathway activation. Box plots of (**A**) deep-learning–generated angioscore and (**B**) phospho-S6 ribosomal protein (PS6) H-score for samples with remaining available slides. Box plot: the lower, middle, and upper edges correspond to the first (Q1), second (Q2; median), and third (Q3) quartiles, respectively. The IQR is defined as Q3−Q1. Whiskers extend to the most extreme data points within 1.5 × IQR below Q1 and above Q3. Data points lying beyond these limits are plotted individually and considered outliers. **P < 0.01, ***P < 0.001, and ****P < 0.0001 by Wilcoxon rank-sum test.

Next, we evaluated angiogenesis. For these studies, we leveraged a deep learning digital pathology platform, which generates an angioscore (21, 25) (see Methods). Using this tool, we found that PM samples had significantly higher angioscores compared with other metastatic sites (*P* < 0.001), which in turn, had lower angioscores than primary tumors (*P* < 0.001) (Figure 6A).

In addition, we evaluated mTORC1 activity. mTORC1 correlates with higher grade, and mTORC1 activation can transform lower grade PBRM1-deficient tumors into higher-grade ccRCC in mouse models (26, 27). As a readout of mTORC1, we used phospho-S6 ribosomal protein (PS6), which has been extensively used as a surrogate (27, 28). We found that PS6 was significantly lower in PM samples compared with primary tumors (*P* = 0.002) and metastases at other sites (*P* < 0.001) (Figure 6B). The difference in PS6 levels was significant even among PBRM1-deficient samples (*P* < 0.001) (Supplemental Figure 6).

**Figure 7:**
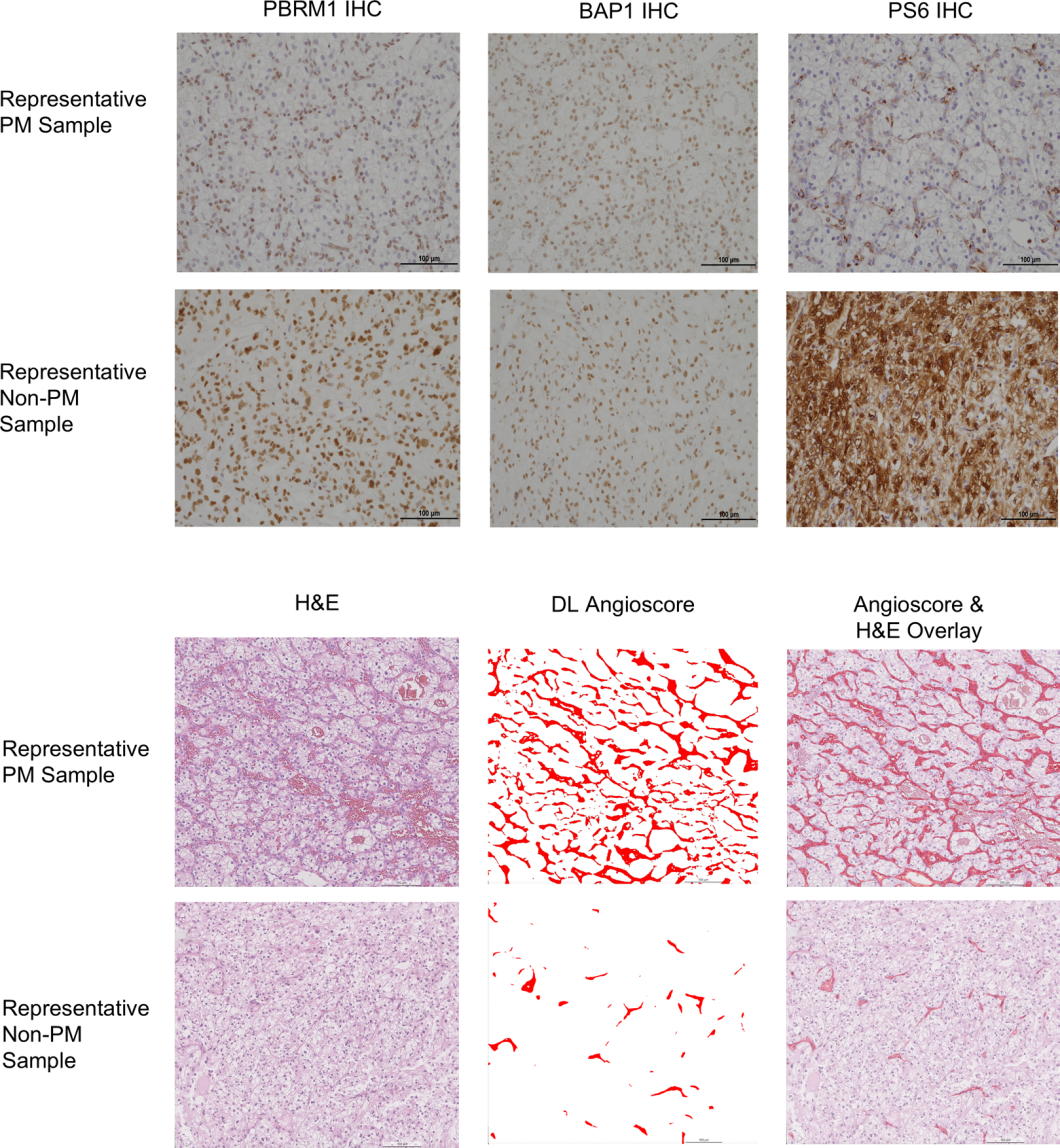
Illustrative images of pancreatic and nonpancreatic metastases. Representative tissue sections of PM and non-PM samples evaluated for PBRM1, BAP1, and phospho-S6 (PS6) by IHC as well as H&E-stained slides with corresponding DL vascular mask and overlay. PM samples are characterized by frequent PBRM1 loss (loss of brown nuclear staining in tumor cells; stromal cells remain positive and serve as a positive control), BAP1^+^, exhibit low mTORC1 (PS6) levels, and high angiogenesis. Scale bars: 100 μm. DL, deep learning; non-PM, nonpancreatic metastasis; PM, pancreatic metastasis.

Overall, these data are consistent with the notion that PMs, in contrast to metastases to other sites, are characterized by highly vascular indolent architectures and lower-grade tumor cells with low mTORC1 scores (Figure 7).

### PM samples arise from indolent clones from primary tumors.

**Figure 8:**
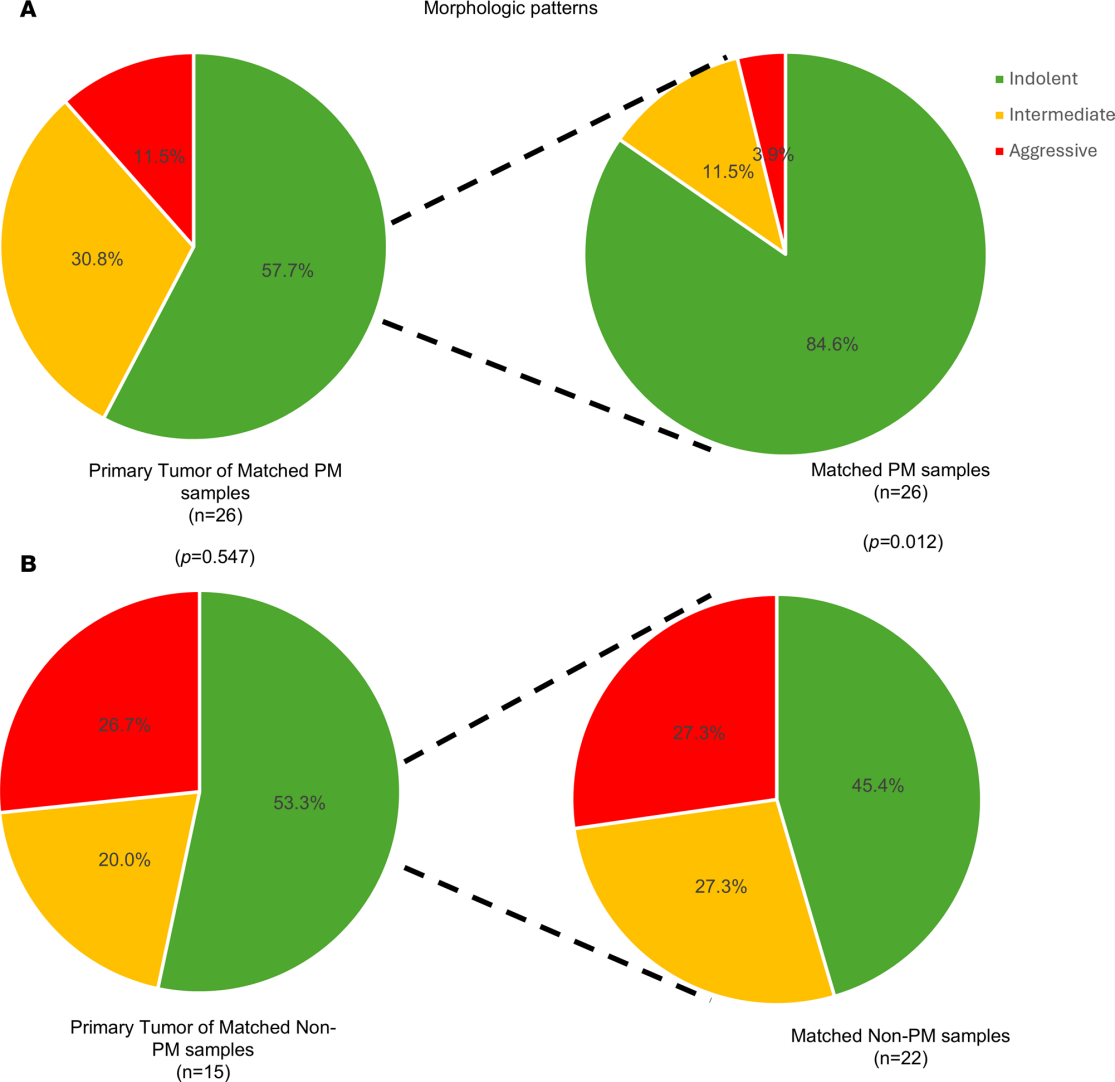
Subgroup analyses of matched primary and metastatic samples. Integrated pie charts of dominant architectural patterns for (**A**) primary tumors and their matched PM samples and (**B**) primary tumors and their matched non-PM samples, compared using the Fisher-Freeman-Halton exact test. Non-PM, nonpancreatic metastasis; PM, pancreatic metastasis.

Overall, these data suggest that PMs, in contrast to metastases to other sites, arise from particularly indolent tumor clones in the primary tumor. To explore this notion further, we analyzed patients with matched primary tumors and metastases. Specifically, we analyzed 26 patients with matched primary tumor and PM samples (26 primary and 26 PM samples) and 15 patients with matched primary and non-PM tumor samples (15 primary and 22 non-PM samples). Of note, 5 patients had samples from primary tumors as well as from both PM and non-PM sites. We started our analyses by evaluating tumor architectures. Although the distribution of architectures in the primary tumors in the 2 cohorts (PM and non-PM) was similar (Figure 8, A and B), a significant difference was observed in tumor architectures between PM samples and samples from other metastatic sites (Figure 8, A and B). Specifically, whereas indolent architectures were observed in 84.6% of PM samples, they were found in only 45.4% of non-PM samples (*P* = 0.012). Conversely, aggressive architectures were more common in non-PM samples (27.3% vs. 3.9%).

Next, we evaluated PBRM1/BAP1/SETD2 status among PM and non-PM samples with matched primary tumors. Specifically, we asked how metastases related to primary tumors with PBRM1 loss. We found that all PMs in patients with primary tumors deficient for PBRM1 were also deficient for PBRM1 (with or without concurrent SETD2 loss). In contrast, the concordance rate between primary tumors and non-PMs was only 73% (*P* = 0.040) (Supplemental Figure 7).

While the foregoing analyses focused on representative samples of primary tumors and metastases obtained from institutions participating in the consortium, we sought to examine the relationship between primary tumors and metastases in greater depth. For these studies, we focused on our University of Texas Southwestern Medical Center (UTSW) cohort of 11 patients with PM and extensive samples available from both the primary tumor and at least 1 metastatic site (see Methods). By systematically analyzing multiple slides, we were able to render a more accurate assessment of the relative frequency of indolent, intermediate, and aggressive architectures in the primary tumor, which were then correlated with findings at metastatic sites.

**Figure 9:**
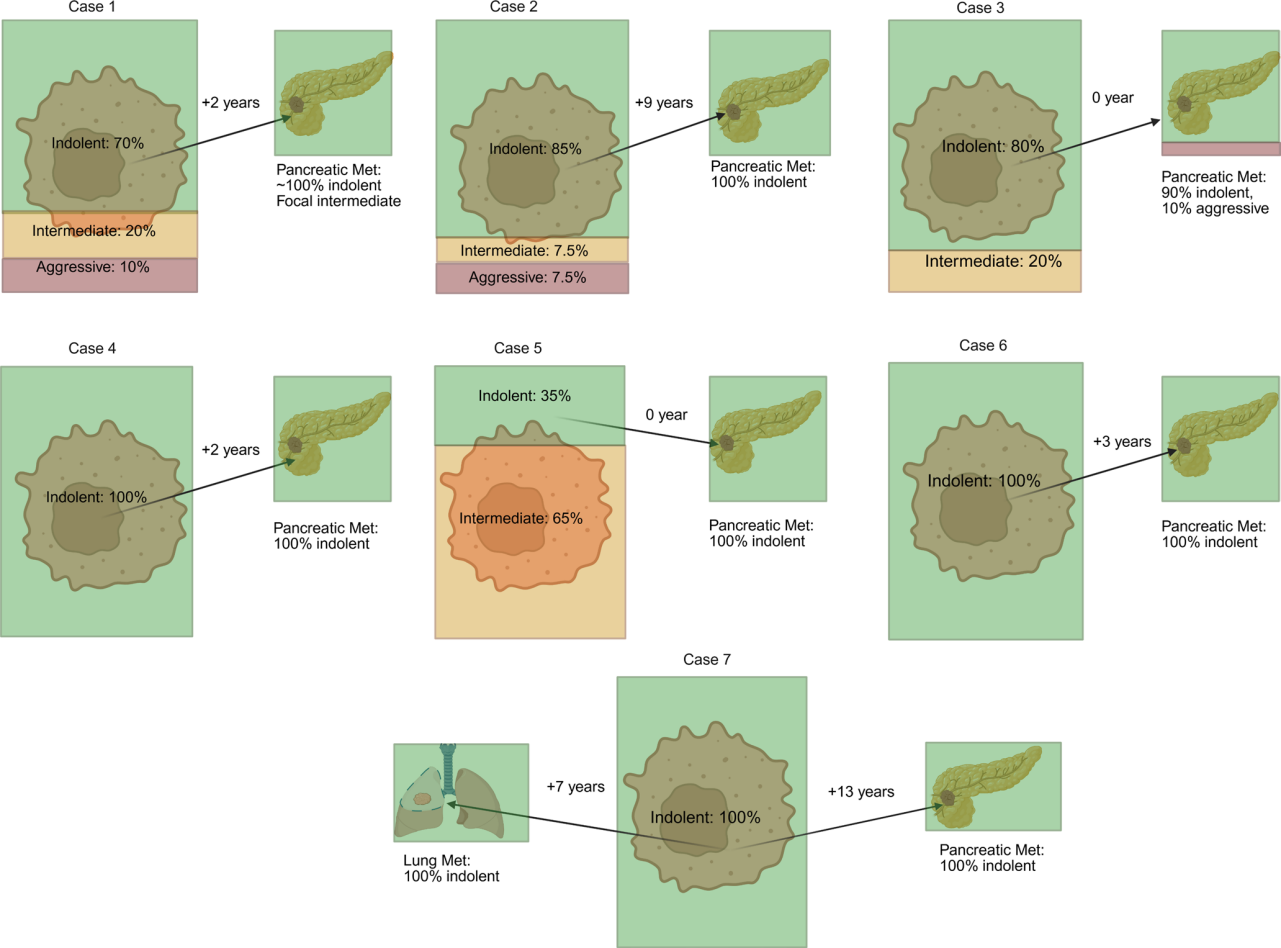
Comprehensive analyses of matched primary and pancreatic metastasis samples. Detailed analyses of intratumoral heterogeneity for UTSW cohort with extensive slides available.

**Figure 10:**
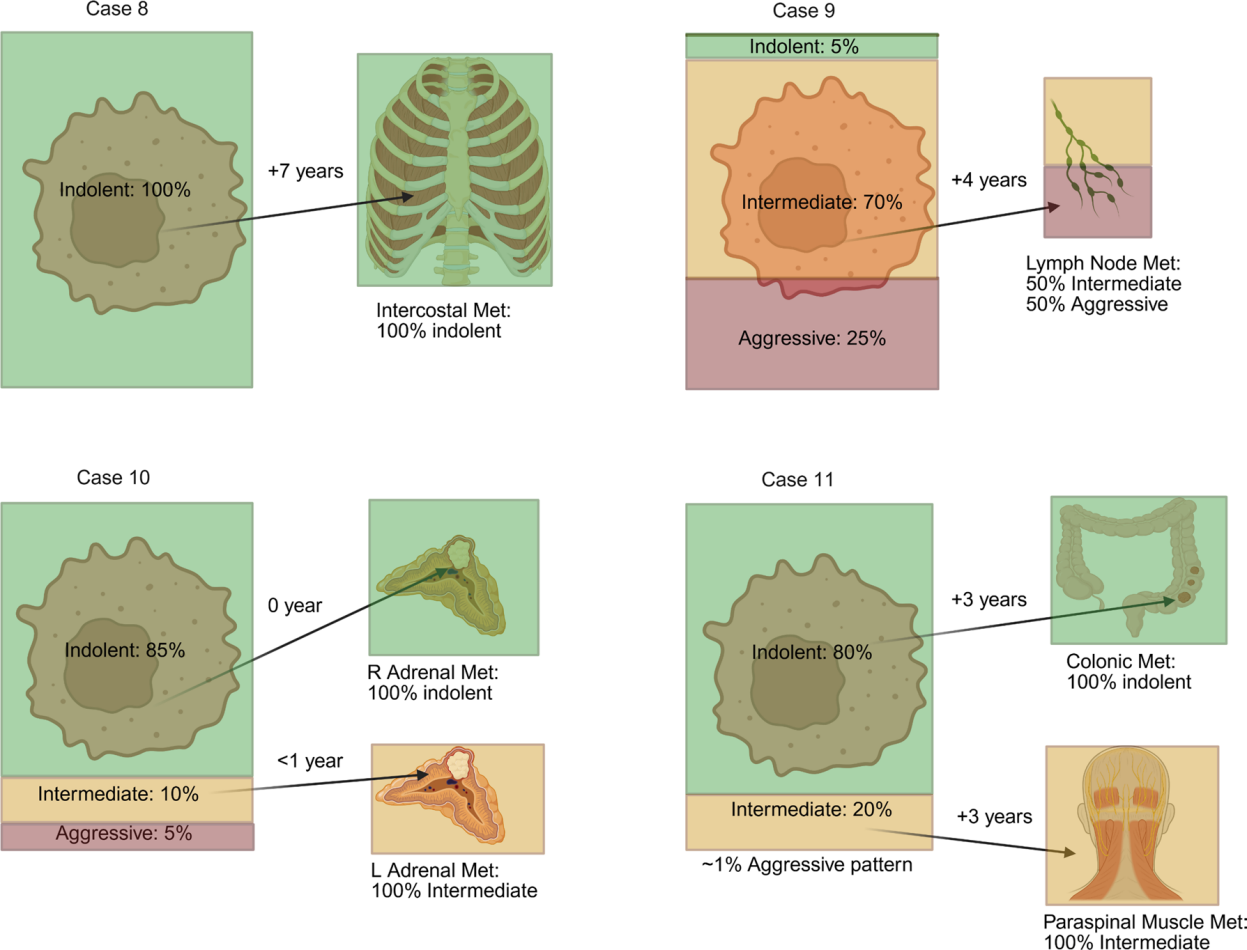
Comprehensive analyses of matched primary and nonpancreatic metastasis samples. Detailed analyses of intratumoral heterogeneity for UTSW cohort with extensive slides available.

As illustrated in Figure 9 and Figure 10, most primary tumors were dominated by indolent architectures. However, only 4/11 primary tumors were homogenously indolent. Intermediate patterns were found in 7/11 tumors and aggressive patterns in 5/11 tumors. These data suggest that while primary tumors that give rise to PMs are dominated by indolent architectures, more aggressive patterns can be identified in comprehensive analyses. In contrast, the same analysis of PMs revealed that the majority of PMs were homogenously indolent (5/7). Thus, despite the identification of intermediate and aggressive architectures in the primary tumor (in 1 case, making up to 65%), PMs were generally indolent. This concept was further explored through multiregional whole-exome sequencing of samples from primary tumors and metastases from 2 patients, including 1 previously reported (29) (Supplemental Figure 8). In Case 1, PMs were characterized by early divergence and included an additional *PBRM1* mutation. Despite being obtained from a surgery 2 years after removal of the primary tumor, these PM samples remained low grade. In contrast, 2 subclones from the primary tumor independently acquired a different *PBRM1* mutation and chromosome 14q gain, progressing to a higher grade. In the second case (Case 4), all primary and PM samples shared 3p loss, as well as *VHL* and *PBRM1* mutations. Overall, the tumor was of low grade, including areas of grade 1 and 2, but 2 PM samples were both of grade 1.

Next, we analyzed non-PM in this cohort with comprehensive histological analyses. Among 7 non-PM metastases tested, 4 were indolent, and 3 were intermediate or aggressive (Figures 9 and 10). Thus, 5/7 PMs were indolent (with the remaining 2 PM being made up of at least 90% indolent features), and only 4/7 non-PMs were indolent. The rate of indolent features in the non-PM samples was higher than expected, but this may reflect the nature of the cohort (i.e., patients with PM) and a sampling bias (with preferential resection of non-PM in patients perceived to have a high likelihood of improved outcomes, such as patients with isolated metastases or long latency periods between the primary tumor and the metastasis).

Overall, these data suggest that PMs are seeded by clones in the primary tumor that are not the most aggressive and are enriched for indolent architectures and low-grade cancer cells.

## Discussion

Prolonged survival of patients with mRCC to the pancreas is well documented in the literature (8, 9, 30). Among patients undergoing pancreatic metastasectomy (patients with only PM), 5-year survival rates from the time of metastasectomy exceed 80% (30). Several reports have shed light on how mRCC with PM represents a specific clade of RCC (9, 10, 31). However, previous studies, including our own, have been limited by low numbers. In this study, we leveraged an international consortium of patients with mRCC with PM (8) and expanded upon previous studies via an interdisciplinary approach that integrates detailed histopathological/digital assessments as well as driver gene/pathway analyses with clinical outcomes. As in the original consortium, of which this subset was representative, we found that patients developing PM exhibited distinct clinicopathological features compared with patients without PM. Patients with PM had more favorable characteristics at initial RCC diagnosis, including earlier disease stage, lower tumor grade, and better IMDC risk scores. Notably, even after controlling for clinicopathological factors, patients with PM had prolonged OS. These findings underscore the unique, indolent natural history of PM.

Further investigation into primary tumors revealed marked differences between PM-associated primary RCC and those without PM. Primary tumors of patients with PM were largely dominated by indolent, highly vascular architectures, and tumor cells were typically of low grade with low mTORC1 pathway activation scores.

Several studies have suggested that the behavior and aggressiveness of RCC can be traced back to the mutational profile. Prior evidence from our lab and others showed that BAP1 loss promotes tumor aggressiveness, whereas PBRM1 loss is linked to more indolent disease (13, 26, 32, 33). Notably, *BAP1* and *PBRM1* mutations anticorrelate in ccRCC, and tracer studies have shown that these mutations occur early during the evolutionary process (15, 32, 33). Furthermore, genetic engineering studies in mice show that *BAP1* and *PBRM1* not only correlate with particular histological features (architecture and grade), but they are indeed drivers of histology and tumor grade (26, 34). Our study shows distinct mutational landscapes between PM and non-PM RCC. Consistent with our earlier report, we found that PM tumors were rarely BAP1 deficient (9). Extending prior observations, we found that PM tumors are frequently PBRM1 deficient. Specifically, PBRM1 loss was observed in 74.3% of primary tumors associated with PM. These rates are substantially higher than previously reported rates in mRCC, which are around 50% (18, 35, 36). Furthermore, even among patients with PM, there was a significantly higher incidence of PBRM1 loss in PM samples compared with metastases at other sites (82.9% vs. 66.4%).

Pathological and multiregional whole-exome sequencing analysis provided additional insight into the evolutionary trajectories of PM. PM samples exhibited more indolent histological profiles compared with non-PM lesions. Specifically, indolent patterns made up 75% of PMs but only 25% of non-PM metastases. In addition, 88.6% of PM were of low grade, compared with 15.4% of non-PM metastases. Given that the non-PM samples used as a reference were from a select group of patients with PM, the differences may have become even more accentuated if the non-PM samples had been taken from patients without PM, who typically have more aggressive primary tumors. Interestingly, PM samples exhibited indolent features despite evidence of more aggressive clones in the primary site. These data are consistent with the notion that the clones that seed the pancreas tend to be particularly indolent. This observation is at odds with the concept that metastases generally arise from particularly aggressive clones. Whole-exome sequencing studies showed that PMs were less evolved than some clones that had not left the primary tumor, which extends previous findings from a handful of patients in the TRACERx consortium (32, 33). Thus, for reasons that remain poorly understood, the pancreas appears to preferentially support the growth of indolent, less evolved, ccRCC clones, which despite their unevolved nature, appear able to gain access to the vasculature, survive in transit, and metastasize to distant sites. In contrast, the pancreatic environment appears to be less supportive of more aggressive clones.

Consistent with prior studies, we found that PMs occur late during the clinical course, adding to evidence from the literature that links PMs to extended metastatic latency (5, 30). Interestingly, a recent study from our group found that at least based on RNA-Seq analyses, latent metastases and PMs tend to cluster together (4), which suggests a shared biology. Thus, a common underlying biology may govern both metastatic latency and pancreatic tropism. In this context, it is interesting to speculate whether indolent, PBRM1-deficient clones are particularly well endowed to survive for prolonged periods without detection.

Our current study suggests that even among patients with PM, PBRM1 is prognostic. Studies by us and others previously showed that PBRM1-deficient RCCs are associated with better outcomes among patients with metastatic disease (13, 18, 19, 37). Given the enrichment for PBRM1 loss among patients with PM, the more frequent loss of PBRM1 (compared with patients without PM) could account for the improved outcome. However, our data show that even among patients with PM, those with PBRM1-deficient tumors, which are the majority, have better survival.

Interestingly, PBRM1 loss may also be predictive of therapy response. Although the original consortium analyses found no difference in survival between patients treated with first line antiangiogenic and IO therapy, our data suggest that in patients with PBRM1-deficient tumors, antiangiogenic therapy is associated with more prolonged disease control. This correlates with histological analyses, which show that PM tumors are characterized not only by frequent PBRM1 loss, but also by heightened angiogenesis. Increased angiogenesis likely underlies the high contrast enhancement that characterizes PM radiologically (38). These results expand upon extensive literature linking PBRM1 loss to angiogenesis and sensitivity to antiangiogenic drugs (20, 21, 39, 40). Analysis of tumors from patients in the IMmotion150 and IMmotion151 clinical trials identified 7 gene expression clusters, and a correlation was found between *PBRM1* alterations and clusters 1 and 2, which were characterized by angiogenesis with enrichment for vascular and VEGF pathway–related genes (20, 21). In addition, molecularly, PBRM1 loss is thought to amplify the HIF response, which involves angiogenesis (41). Nevertheless, at least one study has proposed that PBRM1 loss is associated with better IO response (42). This apparent discrepancy underscores an important observation from our studies, which is that not all PBRM1-deficient tumors behave in the same way. PBRM1 deficient tumors may acquire additional genetic alterations, which may change their original properties. For example, in mouse models, activation of mTORC1 increases the grade of PBRM1-deficient tumors (26). Consistent with this notion, mTORC1 activation among PBRM1-deficient metastases was significantly lower in PMs compared with metastases at other sites. Thus, our data suggest that PMs arise from minimally evolved, low-grade, indolent, PBRM1-deficient clones, which likely retain the original identity. This may explain why these tumors are particularly sensitive to antiangiogenic therapy.

Our study includes patients with isolated PMs as well as those with PMs accompanied by metastases to additional sites. Many of the findings in the overall population remained significant in patients with metastases that extended beyond the pancreas. We did not have enough primary tumors for comprehensive tissue analyses, but we speculate that the PM-only cohort may be characterized by more uniform, indolent, primary tumors, and the cohort with both pancreatic and extrapancreatic metastases may be enriched from primary tumors with greater heterogeneity and more evolved clones.

Our study has several limitations, which include its retrospective nature with its related caveats, including lack of standardized patient follow-up and inadequacies of data abstraction. Despite a large international consortium, numbers were relatively small. In addition, while the cohort with samples was representative of the overall cohort, it remains a subset even if it includes patients from 7 geographically distinct institutions (6 in North America and 1 European site). Another limitation arises from analyses of representative tumor slides, which oversimplifies the complexity (as shown by detailed analyses of our UTSW cohort). With respect to therapy, most patients were treated before the widespread adoption of immune checkpoint inhibitors and many patients receiving IO received IL2 (8). Finally, disease control was inferred from time on therapy, which also has its limitations.

Despite these limitations, these findings support the notion that PMs arise predominantly from indolent clones within primary tumors with distinct molecular and histological features, clinical behavior, and treatment responsiveness. This is striking, particularly in the context of coexisting more aggressive and evolved clones. The characteristics of PM suggest an organ-specific tropism, governed by intrinsic tumor biology with potential for differential therapeutic response.

## Methods

### Sex as a biological variable.

Our study examined men and women, and similar findings are reported for both sexes.

### Study design and patient population.

Tissues were obtained from institutions participating in a previously reported multicenter, international, retrospective study of patients with histologically proven mRCC involving the pancreas (8). All patients in the PM cohort were diagnosed and treated prior to January 2021. Data on patient demographics, tumor characteristics, local and systemic therapy, and outcomes were previously collected using a deidentified template with specific definitions to minimize interobserver variation obtained from the coordinating center (8). Tissues were obtained from patients treated at 7 academic centers, 6 in the United States and 1 in Europe (8). The original study included all histologies, whereas we focused on ccRCC. In addition, the original study evaluated 2 mRCC groups: one with oligometastatic disease to the pancreas (group 1) and another with more broadly metastatic disease treated with systemic therapy (group 2), but there was overlap between the groups and they were combined for this study. To assess how the subset of patients with samples analyzed in this study compared with the originally reported clinical groups, standardized differences were calculated between the subset of patients with tissue and the corresponding original larger groups (8). In addition, how findings from the combined cohort applied to patients with extrapancreatic metastases (group 2) was also evaluated in this study.

For comparison, we used a previously evaluated cohort of patients with metastatic RCC without PM treated with systemic therapy at UTSW (January 2006 until February 2018) (9). The previous report focused on a subset (268 out of 333 patients) for whom IMDC status was available (9), but the full cohort was used here. However, the previous report included all patients with metastatic RCC regardless of histology (9), whereas we focused on those with ccRCC for this study (non-PM reference cohort; *n* = 273). A subset of these patients was previously evaluated for morphological patterns (*n* = 110) (23).

### Pathological analyses.

For all patients, serial unstained slides were requested from a representative clinical block containing the highest graded areas of the primary tumor (*n* = 72). In addition, unstained slides were requested for both PM (*n* = 44) and non-PM samples (*n* = 53). Tissue slides were subjected to H&E staining and IHC. Out of 113 patients for whom tissue and full clinical data were obtained, 108 patients had histologically confirmed ccRCC based on reports by the original institution and central review by an expert genitourinary pathologist, and they are the subject of this study. H&E sections from each sample (primary and/or metastases) were centrally reviewed to assess grade and morphological architecture as previously described (23, 31, 43). Nucleolar grade was assigned to the representative slides from all 72 primary tumors (from 71 patients). The centrally assigned grade was then compared with the grade assigned at the original institution (Supplemental Figure 1). Two patients did not have a reported grade on their deidentified clinical data sheets and were removed from this analysis. One patient had bilateral kidney tumors, and the assigned grade for that patient corresponds to the highest grade observed. Some differences were found, but they were not statistically significant. Most discrepancies involved grade 2 versus grade 3 distinctions, highlighting expected variability in interobserver interpretation and use of Fuhrman’s grading versus contemporary nucleolar grading by centralized review. However, larger variation was noted in 5/69 cases, where the centralized review showed a low grade, but clinical data sheets reported grade 4. One possible explanation is that the unstained slides provided were not from the highest-grade areas of the primary tumor. Nevertheless, there were no statistically significant differences in the distribution of low- and high-grade tumors between the centralized review and the externally reported tumor grade (*P* = 0.233) (Supplemental Figure 1). For patient-level comparisons, tumor grade was based on data reported in the standardized template provided by each participating institution, as primary tumor samples were not available for centralized review for all patients; whereas for tissue-level comparisons, tumor grade was based on the value assigned after centralized review by an expert pathologist blinded to clinical data.

For samples with multiple different architectural patterns, the most aggressive architecture was used to categorize the sample into 1 of 3 categories: indolent (small/large nest, tubular, microcystic), intermediate (trabecular, alveolar, pseudo/papillary), or aggressive (solid, rhabdoid, sarcomatoid). For the UTSW subset of patients in the PM cohort with matched primary and metastasis samples (*n* = 11 patients), more extensive analyses were performed of the primary tumors involving all available tumor-containing H&E-stained slides from different tumor blocks (3–10 slides per primary tumor). For the 11 UTSW patients, each slide was reviewed and assigned a relative frequency of indolent, intermediate, or aggressive patterns. The results were then averaged across all slides available for each tumor. Of note, the most representative pattern was previously reported for 9 of the 11 primary tumors (9), whereas our current review focuses on the most aggressive pattern and the relative frequency of clones in each histological pattern category. Further, we also extended these analyses to include metastatic tumors, which mostly involved a single slide given the smaller tumor volume of metastases.

IHC staining was performed on a representative slide for BAP1, PBRM1, H3K36me3 (a surrogate for SETD2), and PS6 (an indicator of mTORC1 activity) using an Autostainer Link 48 (Dako), as previously described (14, 24, 44). Primary antibodies were obtained from Bethyl Laboratories (PBRM1, 1:4,000 dilution, A301-591A), Santa Cruz Biotechnology (BAP1, 1:700 dilution, sc-28383), Active Motif (H3K36me3, 1:4,500 dilution, 61021), and Cell Signaling Technology (PS6, 1:300 dilution, 5364S). Appropriate positive and negative controls were incorporated in each IHC run. All stains were scored without knowledge of the associated clinical information. For BAP1, PBRM1, and H3K36me3, tumors were characterized as negative (loss of the protein/marker) when they lacked nuclear staining in the presence of positive staining in intratumoral lymphocytes and stromal cells that served as an internal positive control. Cases lacking staining of intratumoral lymphocytes and stromal cells were considered uninterpretable and were removed from analyses. Samples with heterogeneous/focal loss were categorized as being overall negative for that tumor suppressor protein. For patients with different staining patterns across samples (primary tumor vs. metastasis), the patient was categorized to have loss if any sample showed unequivocal protein loss. PS6 staining was quantified using an H-score (45). Briefly, PS6 staining intensity was scored as 0 (negative), 1 (weak), 2 (moderate), or 3 (strong) and was multiplied by the percentage of tumor cells positive for the stain.

### Digital pathology.

For digital pathology analyses, we applied the following pipeline. Tumor regions were identified on H&E slides using a deep learning region classification model (25). These regions were manually reviewed for correctness and if needed, further refined through manual annotation. Within the identified tumor regions, an image-based angioscore was predicted using a deep learning model designed to emulate an RNA-based angioscore (25). When derived from RNA, the angiogenesis score is quantitated based on the expression of 6 angiogenesis marker genes (*VEGFA*, *KDR*, *ESM1*, *PECAM1*, *ANGPTL4*, and *CD34*) (21). In contrast, the deep learning–derived vascular mask was used to quantify the proportion of pixels corresponding to vasculature. The computed vascular proportion was designated as the angioscore for the slide.

### Construction of genetic phylogenic trees.

Multiregional whole-exome sequencing was performed on FFPE tissue samples from 2 UTSW patients with ccRCC with PM. Sample collection, somatic variant and indel calling, copy number analysis, and phylogenetic tree construction were carried out as described previously (29). In brief, tumors and metastases were sampled using punch biopsies to capture tumor heterogeneity. High-confidence somatic single nucleotide variants and indels were identified using a consensus of MuTect2 (46), FreeBayes (47), and Strelka2 (48). Copy number alterations were inferred using FACETS and FACETS-suite (49). Phylogenetic trees were constructed based on mutation and arm-level copy number data using Hamming distance matrices and nearest-neighbor algorithms optimized by maximum parsimony, rooted to a corresponding normal sample. COSMIC Cancer Gene Census (50) and TRACERx Renal signature copy number variations (33) were used as a reference for the annotation.

### Clinical endpoints.

Clinical outcomes were obtained from the coordinating center using a deidentified data collection template based on the original report with a cutoff date of January 16, 2021 (8). Median OS was analyzed from the time of initial metastasis diagnosis until death or last documented follow-up. TOT with first-line therapy was defined as time from initiation of systemic therapy to discontinuation for any reason (i.e., progressive disease, toxicity, patient or practitioner preference, or death). Historically, the discontinuation rate for angiogenesis inhibitors due to adverse events ranges from 4% to 24% (51–54) and is similar to the 8%–22% observed with IO agents (54–56). With the assumption that most patients discontinued therapy due to disease progression (or death), TOT was used to approximate progression-free survival, as in the original report (9).

### Statistics.

Baseline clinicopathological characteristics were tabulated across both the PM and reference cohorts and compared. Categorical variables were compared using a Fisher’s exact test for 2×2 tables and the Fisher-Freeman-Halton exact test for larger contingency tables. Continuous variables were compared using the Wilcoxon rank-sum test. For OS, the survival probability was calculated by the Kaplan-Meier method starting from time of initial metastasis. TOT also deployed the Kaplan-Meier method. Time-to-event results are reported with HR, 95% CI for the HR, and *P* value from Cox regression analyses. Statistical analyses were conducted using SPSS version 25.0 (IBM) and RStudio, version 2024.12.1+563 to 2026.01.0+392. *P* values are 2-sided, with statistical significance defined as *P* less than 0.05.

### Study approval.

IRB approval (or exemption) was obtained at each institution, including UTSW; University of Colorado, Aurora, Colorado, USA; Moores Cancer Center, UCSD; University of Utah; The Ohio State University; Medical Oncology & Therapeutics Research, City of Hope; and University Hospitals Leuven, KU Leuven. IRB approvals were obtained that waived the need for informed consent because of minimal risk, based on retrospective review of existing data.

### Data availability.

Values for all data points in graphs are reported in the Supporting Data Values file. Whole-exome sequencing results for Case 1/KC01888 were previously published and can be accessed at European Genome-Phenome Archive (EGA) with the EGA ID EGAS50000001064 (29). The patient in Case 4 did not explicitly consent to the release of genomic data.

## Author contributions

HX was responsible for data curation, formal analysis, methodology, project administration, validation, writing of the original draft, and review and editing. PK contributed to the pathologic evaluation, methodology, resources, supervision, validation, and review and editing. AC, AWN, and SR were responsible for data curation, formal analysis, and review and editing. OB, CS, and VT were responsible for project administration. RE contributed to data curation, project administration, and review and editing. AP, JM, CD, AVB, JP, HL, DZ, LM, BB, HZ, and CR were responsible for data curation. KAC performed data curation and review and editing. NA, AM, RM, and ETL contributed to supervision and review and editing. SP supervised the project. JB was responsible for conceptualization, funding acquisition, methodology, resources, supervision, and review and editing. The order of co–first authorship was determined by the time spent coordinating and administering the project.

## Funding support

This work is the result of NIH funding, in whole or in part, and is subject to the NIH Public Access Policy. Through acceptance of this federal funding, the NIH has been given a right to make the work publicly available in PubMed Central.

- NIH Kidney Cancer SPORE grant (P50CA196516).
- The Cancer Prevention and Research Institute of Texas (RP220294).
- Jan and Bob Pickens Distinguished Professorship in Medical Science endowment.
- Brock Fund for Medical Science Chair in Pathology.

## Supplementary Material

Supplemental data

ICMJE disclosure forms

Supporting data values

## Figures and Tables

**Table 1:**
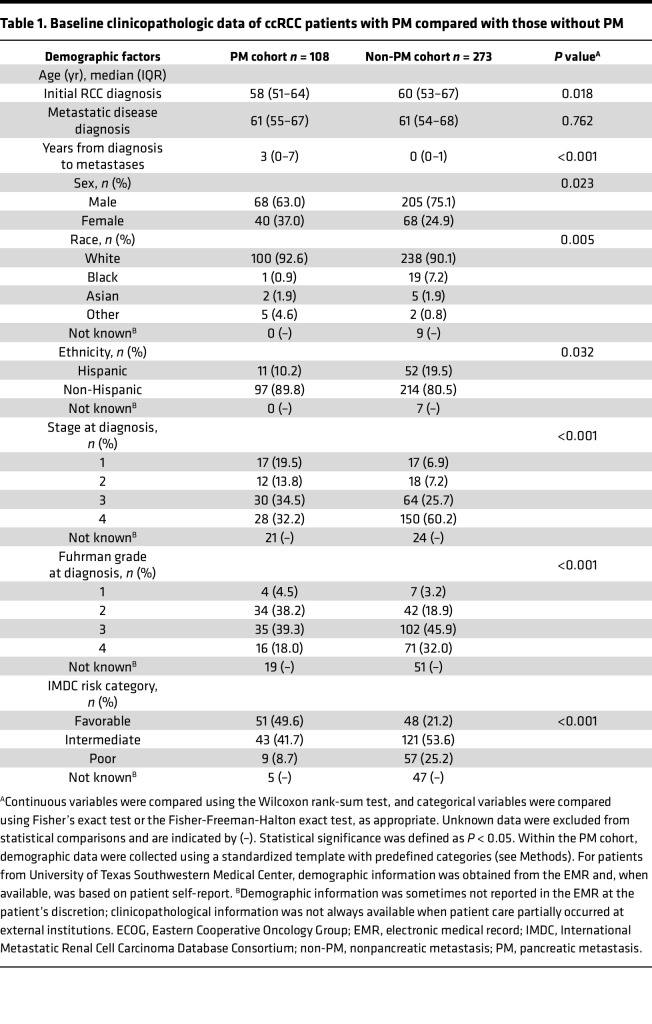
Baseline clinicopathologic data of ccRCC patients with PM compared with those without PM

**Table 2:**
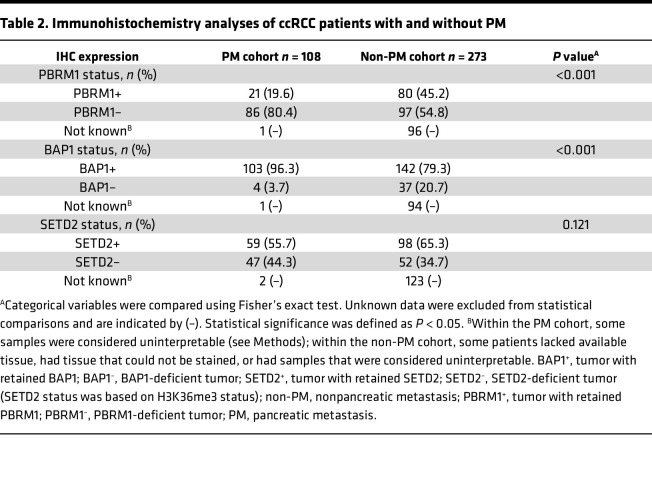
Immunohistochemistry analyses of ccRCC patients with and without PM

**Table 3:**
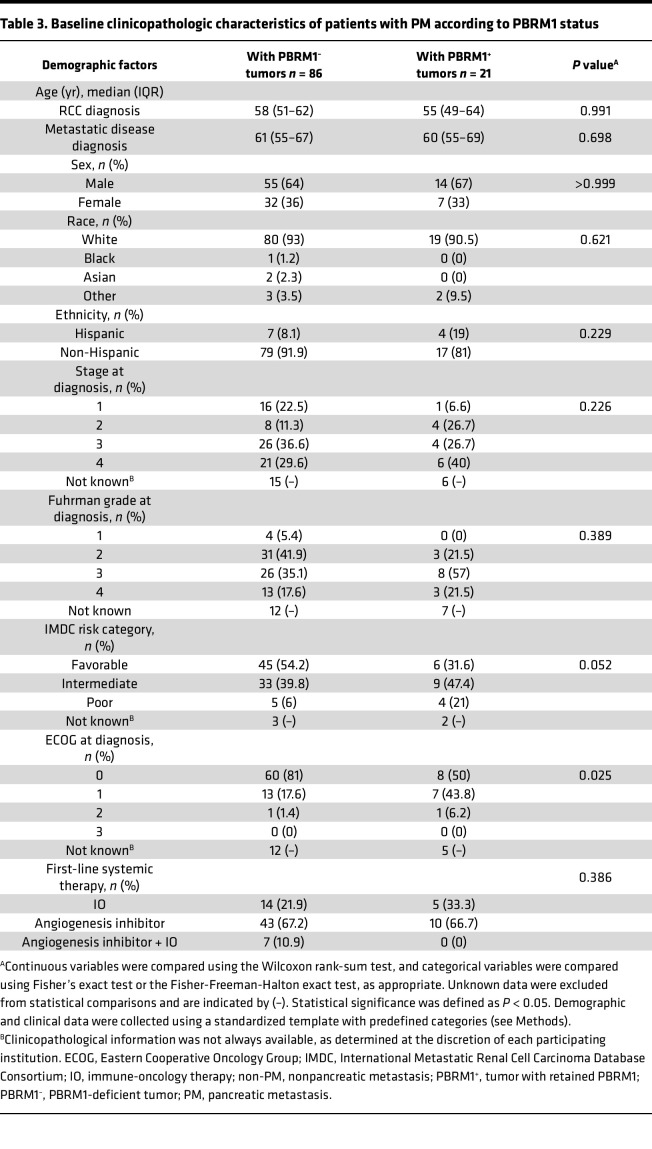
Baseline clinicopathologic characteristics of patients with PM according to PBRM1 status

## Version 1 03/09/2026

Electronic publication
