# Supplementary material for: Determinants of pancreatic tropism in metastatic renal cell carcinoma
Source: JCI Insight. 2026 Mar 9;11(5):e195195. doi: 10.1172/jci.insight.195195 (PMC13041672; doi:10.1172/jci.insight.195195)
Supplement: Supplemental data [file jciinsight-11-195195-s252.pdf]

Supplementary Table 1

Demographic Factors	Subset with samples (Group 1) N=38	PM Consortium (Group 1) N=87	Standardized difference
**Sex, n (%)**			
Male	23 (60.5)	46 (52.9)	0.15
Female	15 (39.5)	41 (47.1)	
**Race, n (%)**			
White	38 (100)	79 (90.8)	0.45
Black	0 (0)	4 (4.6)	
Asian	0 (0)	1 (1.1)	
Other	0 (0)	3 (3.4)	
**Ethnicity, n (%)**			
Hispanic	3 (7.9)	8 (9.2)	0.05
Non-Hispanic	35 (92.1)	79 (90.8)	
**Stage at Diagnosis, n (%)**			
1	6 (20.7)	16 (23.9)	0.26
2	6 (20.7)	20 (29.9)	
3	11 (37.9)	19 (28.4)	
4	6 (20.7)	12 (17.9)	
Unknown	9 (-)	20 (-)	
**Histology, n (%)**			0
Clear Cell	38 (100)	86 (100)	
Not known	0 (-)	1 (-)	
**MSKCC Risk Category, n (%)**			
Favorable	17 (48.6)	44 (53.0)	0.21
Intermediate	16 (45.7)	37 (44.6)	
Poor	2 (5.7)	2 (2.4)	
Unknown	3 (-)	4 (-)	
**ECOG at Diagnosis, n (%)**			
0	27 (81.8)	68 (91.9)	0.23
1	6 (18.2)	6 (8.1)	
2	0 (–)	0 (–)	
3	0 (–)	0 (–)	
Unknown	5 (-)	13 (–)	
**IMDC Risk Category, n (%)**			
Favorable	23 (63.9)	50 (59.5)	0.22
Intermediate	11 (30.6)	32 (38.1)	
Poor	2 (5.5)	2 (2.4)	
Unknown	2 (-)	3 (-)	

Supplementary Table 2

Demographic Factors	Subset with samples (Group 2) N=77	PM Consortium (Group 2) N=208	Standardized difference
**Sex, n (%)**			
Male	48 (62.3)	133 (63.9)	0.03
Female	29 (37.7)	75 (36.1)	
**Race, n (%)**			
White	69 (89.6)	187 (89.9)	0.09
Black	1 (1.3)	2 (1.0)	
Asian	2 (2.6)	8 (3.8)	
Other	5 (6.5)	11 (5.3)	
**Ethnicity, n (%)**			
Hispanic	9 (11.7)	26 (12.5)	0.03
Non-Hispanic	68 (88.3)	182 (87.5)	
**Stage at Diagnosis, n (%)**			
1	12 (19.0)	26 (17.9)	0.11
2	8 (12.7)	22 (15.2)	
3	20 (31.7)	40 (27.6)	
4	23 (36.6)	57 (39.3)	
Unknown	14 (-)	63 (-)	
**Histology, n (%)**			
Clear Cell	77 (100)	193 (92.8)	0.39
**ECOG at Diagnosis, n (%)**			
0	47 (74.6)	126 (75.4)	0.16
1	14 (22.2)	34 (20.4)	
2	2 (3.2)	5 (3.0)	
3	0 (0)	2 (1.2)	
Unknown	14 (-)	41 (-)	
**IMDC Risk Category, n (%)**			
Favorable	34 (46.0)	65 (36.5)	0.18
Intermediate	32 (43.2)	89 (50)	
Poor	8 (10.8)	24 (13.5)	
Unknown	3 (-)	30 (-)	
**Treatment Subgroup, n (%)**			
Angiogenesis inhibitor	51 (66.2)	151 (72.6)	0.14
IO	19 (24.7)	39 (18.7)	
Angiogenesis inhibitor + IO	7 (9.1)	18 (8.7)	
**Local treatment**			
No	56 (72.7)	177 (85.1)	0.31
Yes	21 (27.3)	31 (14.9)	

Supplementary Table 3

Demographic Factors	PM Cohort with Extrapancreatic Metastases N=77	Non-PM Cohort N=273	P value
**Age (yr), median (IQR)**			
Initial RCC diagnosis	58 (50 - 65)	60 (53 - 67)	
Years from diagnosis to metastases	2 (0 – 6)	0 (0 – 1)	<0.001
**Sex, n (%)**			
Male	48 (62.3)	205 (75.1)	0.043
Female	29 (37.7)	68 (24.9)	
**Race, n (%)**			
White	69 (89.6)	238 (90.1)	0.223
Black	1 (1.3)	19 (7.2)	
Asian	2 (2.3)	5 (1.9)	
Other	5 (6.5)	2 (0.8)	
Not Known	0 (-)	9 (-)	
**Ethnicity, n (%)**			
Hispanic	9 (11.7)	52 (19.5)	0.129
Non-Hispanic	68 (88.3)	214 (80.5)	
Not Known	0 (0)	7 (-)	
**Stage at Diagnosis, n (%)**			
1	12 (19.0)	17 (6.9)	0.001
2	8 (12.7)	18 (7.2)	
3	20 (31.7)	64 (25.7)	
4	23 (36.6)	150 (60.2)	
Unknown	14 (-)	24 (-)	
**Fuhrman Grade at Diagnosis, n (%)**			
1	2 (3.1)	7 (3.2)	0.018
2	24 (36.9)	42 (18.9)	
3	25 (38.5)	102 (45.9)	
4	14 (21.5)	71 (32.0)	
Unknown	12 (-)	51 (-)	
**IMDC Risk Category, n (%)**			
Favorable	34 (46.0)	48 (21.2)	<0.001
Intermediate	32 (43.2)	121 (53.6)	
Poor	8 (10.8)	57 (25.2)	
Unknown	3 (-)	47 (-)	

Supplementary Table 3

Demographic Factors	PM Cohort with Extrapancreatic Metastases N=77	Non-PM Cohort N=273	P value
**Age (yr), median (IQR)**			
Initial RCC diagnosis	58 (50 - 65)	60 (53 - 67)	
Years from diagnosis to metastases	2 (0 – 6)	0 (0 – 1)	<0.001
**Sex, n (%)**			
Male	48 (62.3)	205 (75.1)	0.043
Female	29 (37.7)	68 (24.9)	
**Race, n (%)**			
White	69 (89.6)	238 (90.1)	0.223
Black	1 (1.3)	19 (7.2)	
Asian	2 (2.3)	5 (1.9)	
Other	5 (6.5)	2 (0.8)	
Not Known	0 (-)	9 (-)	
**Ethnicity, n (%)**			
Hispanic	9 (11.7)	52 (19.5)	0.129
Non-Hispanic	68 (88.3)	214 (80.5)	
Not Known	0 (0)	7 (-)	
**Stage at Diagnosis, n (%)**			
1	12 (19.0)	17 (6.9)	0.001
2	8 (12.7)	18 (7.2)	
3	20 (31.7)	64 (25.7)	
4	23 (36.6)	150 (60.2)	
Unknown	14 (-)	24 (-)	
**Fuhrman Grade at Diagnosis, n (%)**			
1	2 (3.1)	7 (3.2)	0.018
2	24 (36.9)	42 (18.9)	
3	25 (38.5)	102 (45.9)	
4	14 (21.5)	71 (32.0)	
Unknown	12 (-)	51 (-)	
**IMDC Risk Category, n (%)**			
Favorable	34 (46.0)	48 (21.2)	<0.001
Intermediate	32 (43.2)	121 (53.6)	
Poor	8 (10.8)	57 (25.2)	
Unknown	3 (-)	47 (-)	

Supplementary Table legends

Supplementary Table 1. Representativity analysis of PM patients with samples corresponding to the PM Consortium Group 1. Demographic and clinical data were collected using a standardized template with predefined categories (see Methods). Clinicopathologic information was not always available, as determined at the discretion of each participating institution. (-), Missing information excluded from analyses; ECOG, Eastern Cooperative Oncology Group; IMDC, International Metastatic Renal Cell Carcinoma Database Consortium; MSKCC, Memorial Sloan-Kettering Cancer Center; PM, pancreatic metastases.

Supplementary Table 2. Representativity analysis of PM patients with samples corresponding to the PM Consortium Group 2. Demographic and clinical data were collected using a standardized template with predefined categories (see Methods). Clinicopathologic information was not always available, as determined at the discretion of each participating institution. (-), Missing information excluded from analyses; ECOG, Eastern Cooperative Oncology Group; IMDC, International Metastatic Renal Cell Carcinoma Database Consortium; IO, immuneoncology therapy; PM, pancreatic metastases.

Supplementary Table 3. Baseline clinicopathologic data of ccRCC patients from the previously published PM Consortium who had metastasis to pancreas plus additional sites, compared to metastatic patients without PM. Demographic data were collected using a standardized template with predefined categories (see Methods). For patients from UT Southwestern, demographic information was obtained from the electronic medical record and, when available, was based on patient self-reports. Demographic information was sometimes not reported in the EMR at the patient's discretion; clinicopathologic information was not always available when patient care partially occurred at external institutions. (-), Missing information excluded from analyses; IMDC, International Metastatic Renal Cell Carcinoma Database Consortium; Non-PM, Non-pancreatic metastases; PM, pancreatic metastases.

Supplementary Figure 1

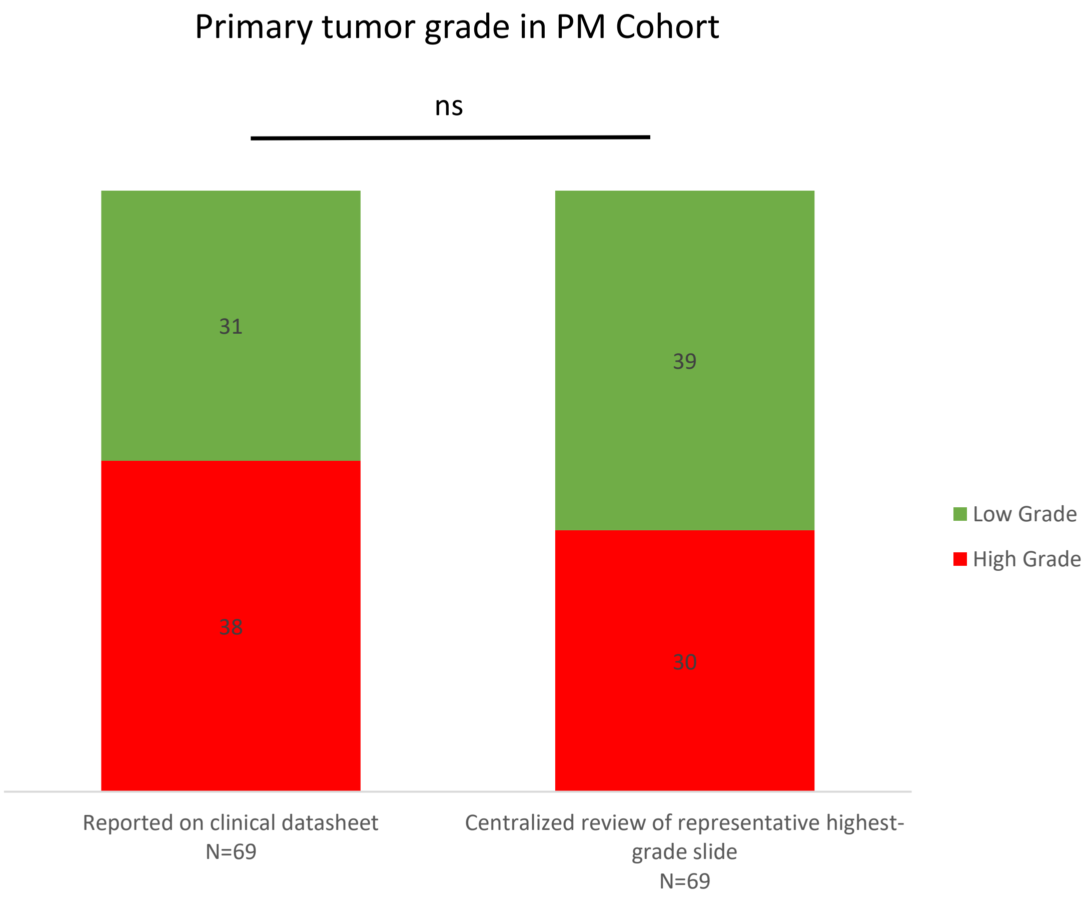

Supplementary Figure 2

Multiple Regression	Reference	Comparison	Overall Survival HR (95% CI)
PM Status	No PM	Presence of PM	0.38 (0.24 – 0.61)
Sex	Male	Female	0.86 (0.58 – 1.27)
Race	White	Non-White	0.99 (0.55 – 1.78)
Ethnicity	Hispanic	Non-Hispanic	0.78 (0.50 – 1.23)
IMDC at Diagnosis	Low Risk	Intermediate Risk	1.64 (1.03 – 2.63)
	Low Risk	Poor Risk	4.43 (2.56 – 7.67)
Stage at Diagnosis	Stage 1	Stage 2	0.59 (0.24 – 1.45)
	Stage 1	Stage 3	0.74 (0.38 – 1.44)
	Stage 1	Stage 4	0.86 (0.45 – 1.64)
Grade at Diagnosis	Grade 1	Grade 2	0.79 (0.26 – 2.36)
	Grade 1	Grade 3	1.37 (0.47 – 3.96)
	Grade 1	Grade 4	1.30 (0.44 – 3.81)

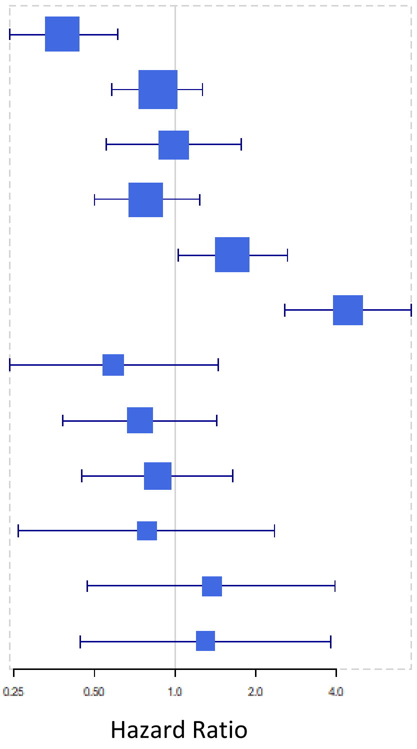

Supplementary Figure 3

			Overall Survival
**Multiple Regression**	**Reference**	**Comparison**	**HR (95% CI)**
PBRM1 + ECOG	PBRM1 +	PBRM1 -	0.35 (0.17 – 0.74)
	Score 0	Score 1	1.64 (0.76 – 3.54)
	Score 0	Score 2	7.84 (1.75 – 35.21)
PBRM1 + IMDC	PBRM1 +	PBRM1 -	0.36 (0.18 – 0.73)
	Low Risk	Intermediate Risk	1.24 (0.60 – 2.55)
	Low Risk	Poor Risk	3.04 (1.09 – 8.50)

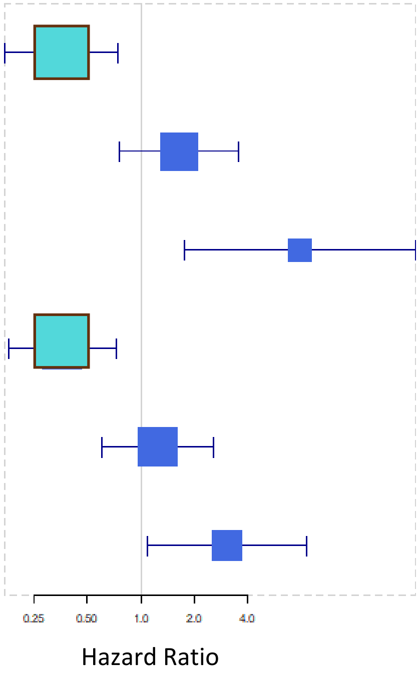

Supplementary Figure 4

A

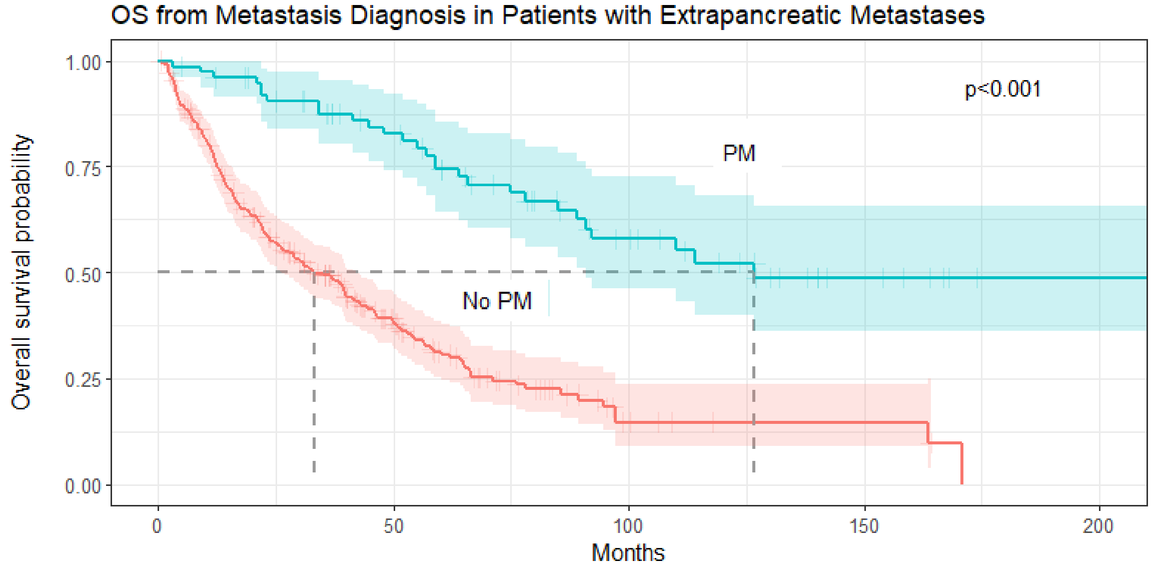

No PM						
	At Risk	273	63	7	3	0
	Events	0	151	178	178	180
PM						
	At Risk	76	51	24	7	1
	Events	0	12	25	28	28

	**PM**	**No PM**
Median OS	127 (91, NR) mos.	33 (26, 42) mos.
HR (95% CI)	0.26 (0.17 – 0.39)	Reference
p-value	<0.001	Reference

B

Overall Survival

**Multiple Regression**	**Reference**	**Comparison**	**HR (95% CI)**
PM Status	No PM	Presence of PM	0.32 (0.19 – 0.54)
Sex	Male	Female	0.90 (0.60 – 1.34)
Race	Non-White	White	0.99 (0.55 – 1.79)
IMDC at Diagnosis	Low Risk	Intermediate Risk	1.67 (1.00 – 2.69)
	Low Risk	Poor Risk	4.67 (2.61 – 8.39)
Stage at Diagnosis	Stage 1	Stage 2	0.56 (0.23 – 1.46)
	Stage 1	Stage 3	0.70 (0.35 – 1.42)
	Stage 1	Stage 4	0.78 (0.39 – 1.57)
Grade at Diagnosis	Grade 1	Grade 2	1.48 (0.34 – 6.50)
	Grade 1	Grade 3	2.20 (0.51 – 9.43)
	Grade 1	Grade 4	2.31 (0.54 – 9.98)

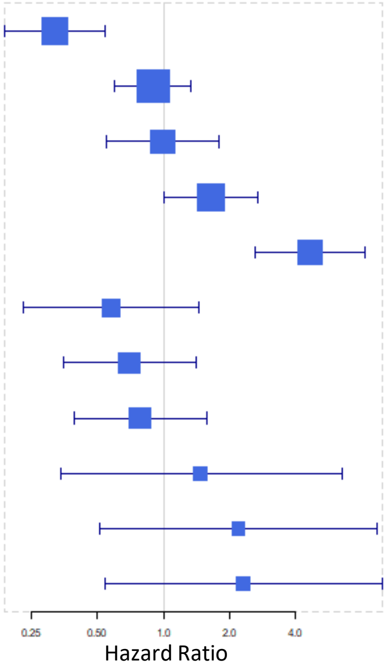

Supplementary Figure 5

A

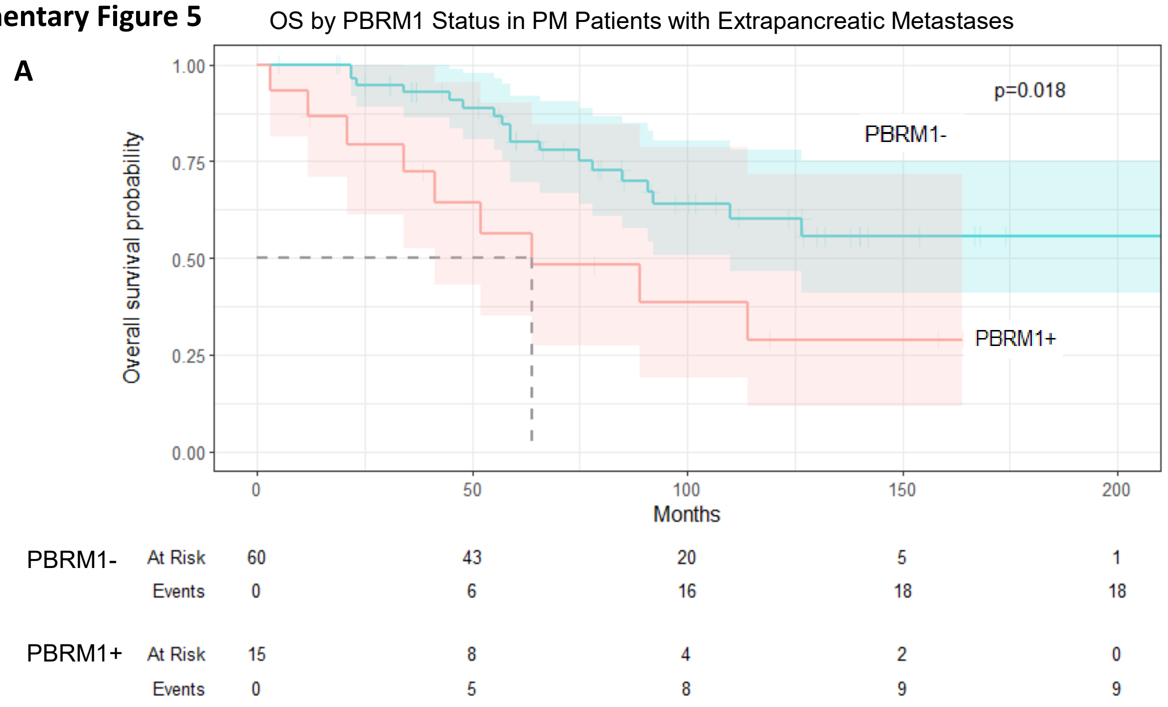

	PBRM1 -	PBRM1 +
Median OS	NR (110, NR) mos.	64 (41, NR) mos.
HR (95% CI)	0.39 (0.18 – 0.88)	Reference
p-value	0.022	Reference

B

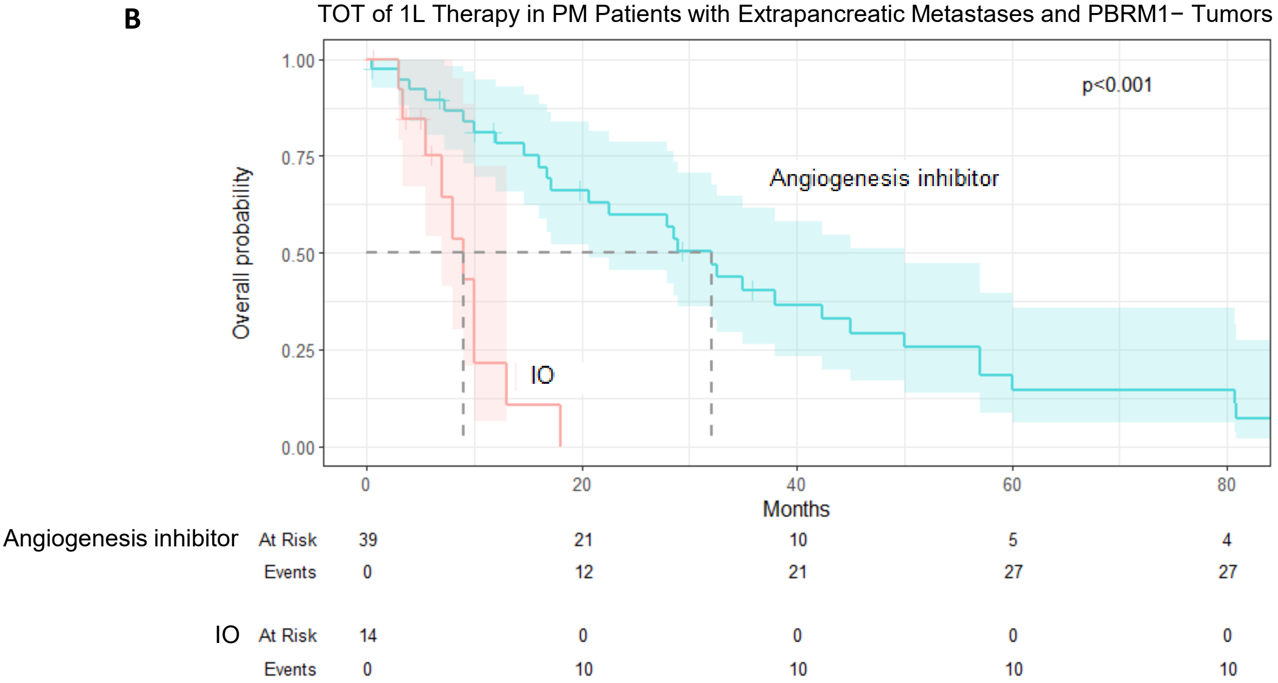

	Angiogenesis inhibitor	IO
Median OS	32 (21, 50) mos.	9.1 (7, NR) mos.
HR (95% CI)	0.16 (0.07 – 0.40)	Reference
p-value	<0.001	Reference

Supplementary Figure 6

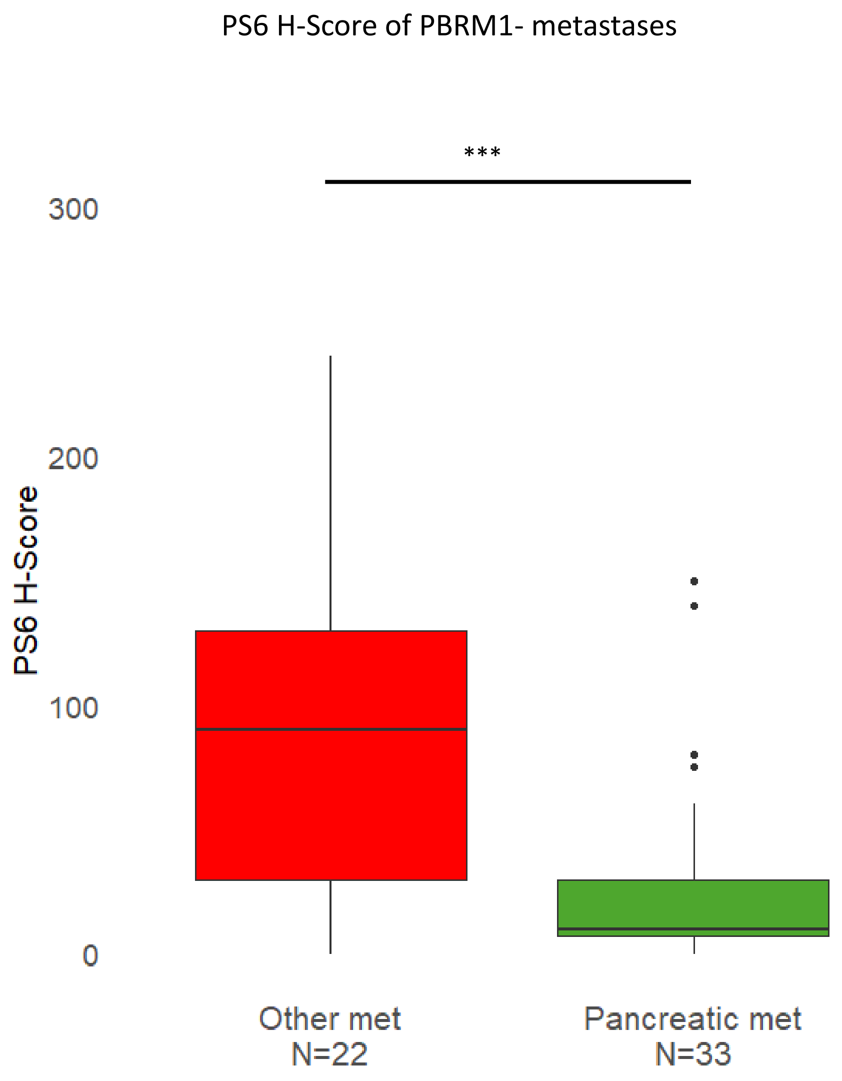

Supplementary Figure 7

Matched PM with PBRM1-deficient Primary, N=17

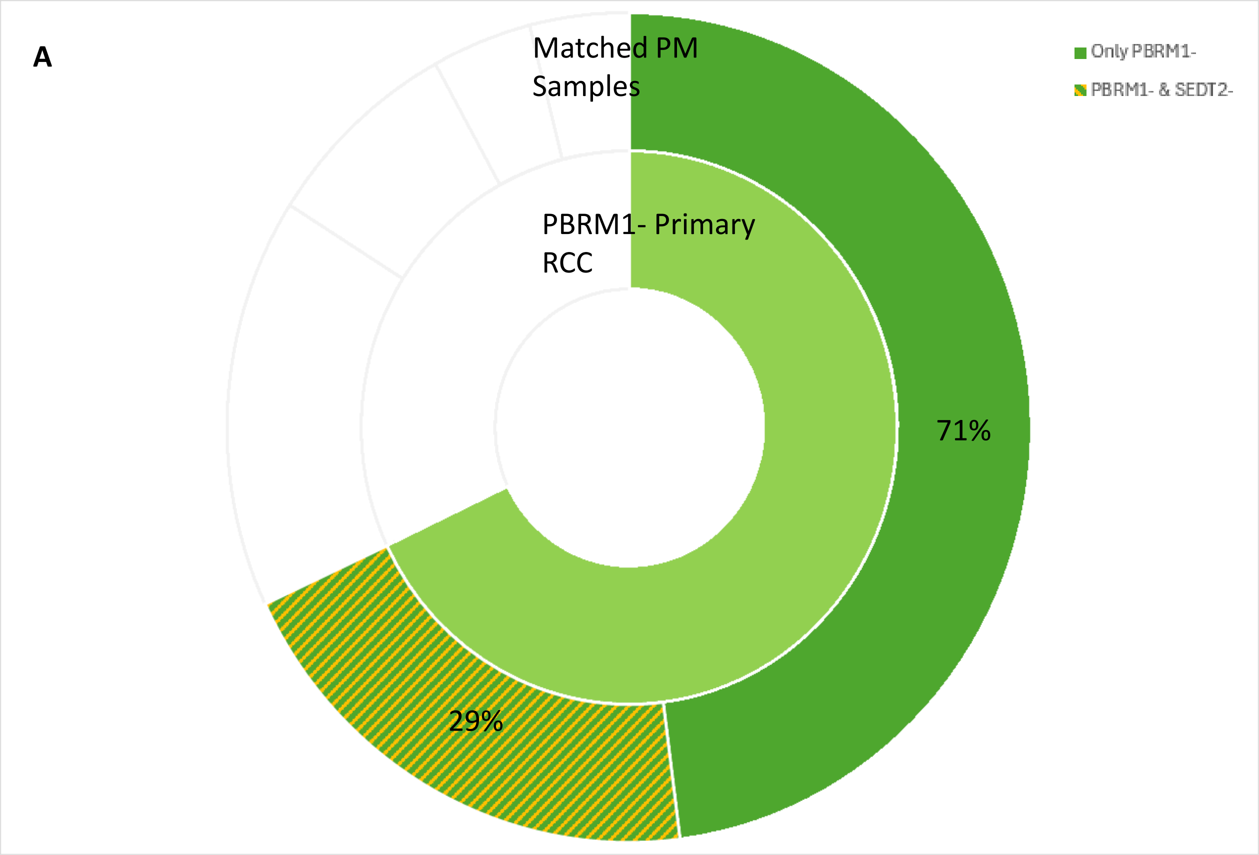

Matched non-PM with PBRM1-deficient Primary, N=11

P=0.040

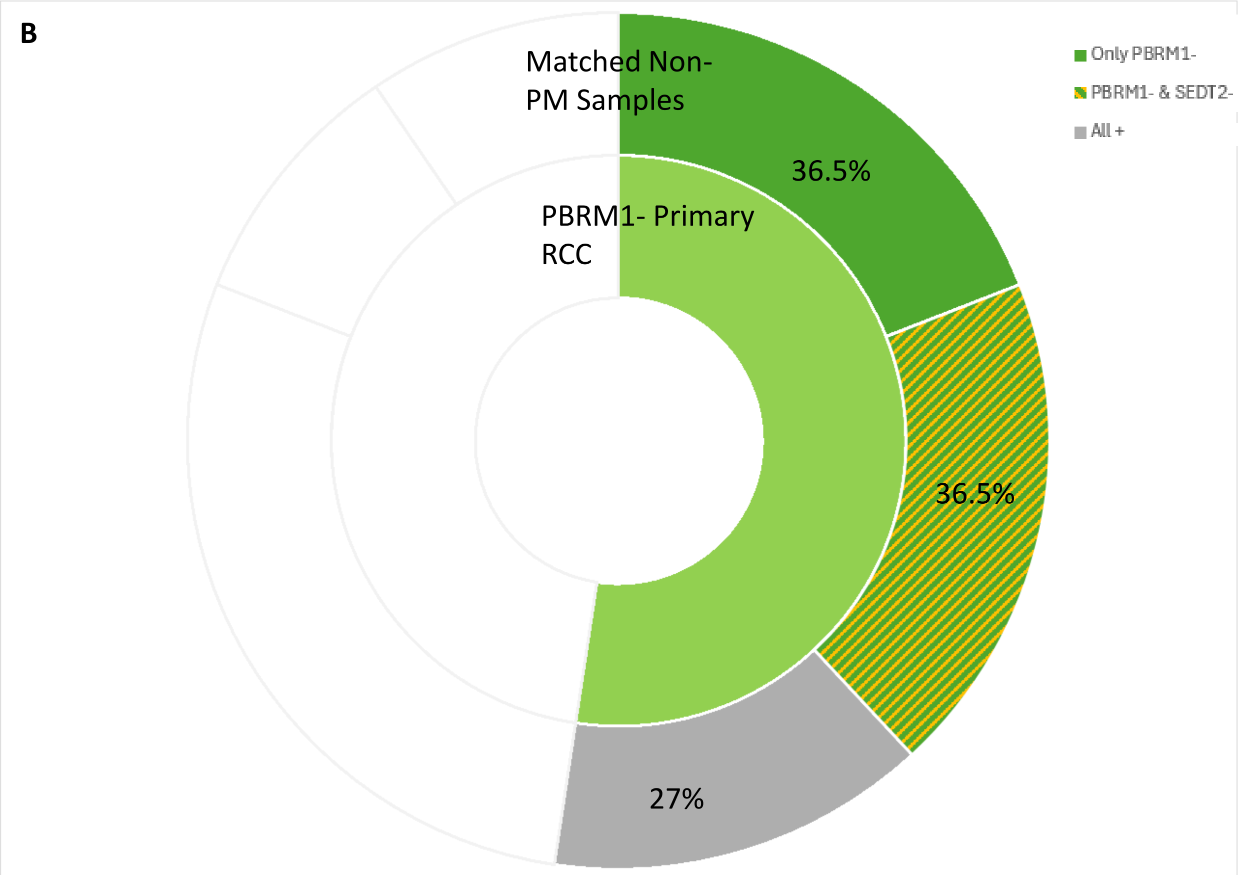

Supplementary Figure 8

Case 1 (KC01888)

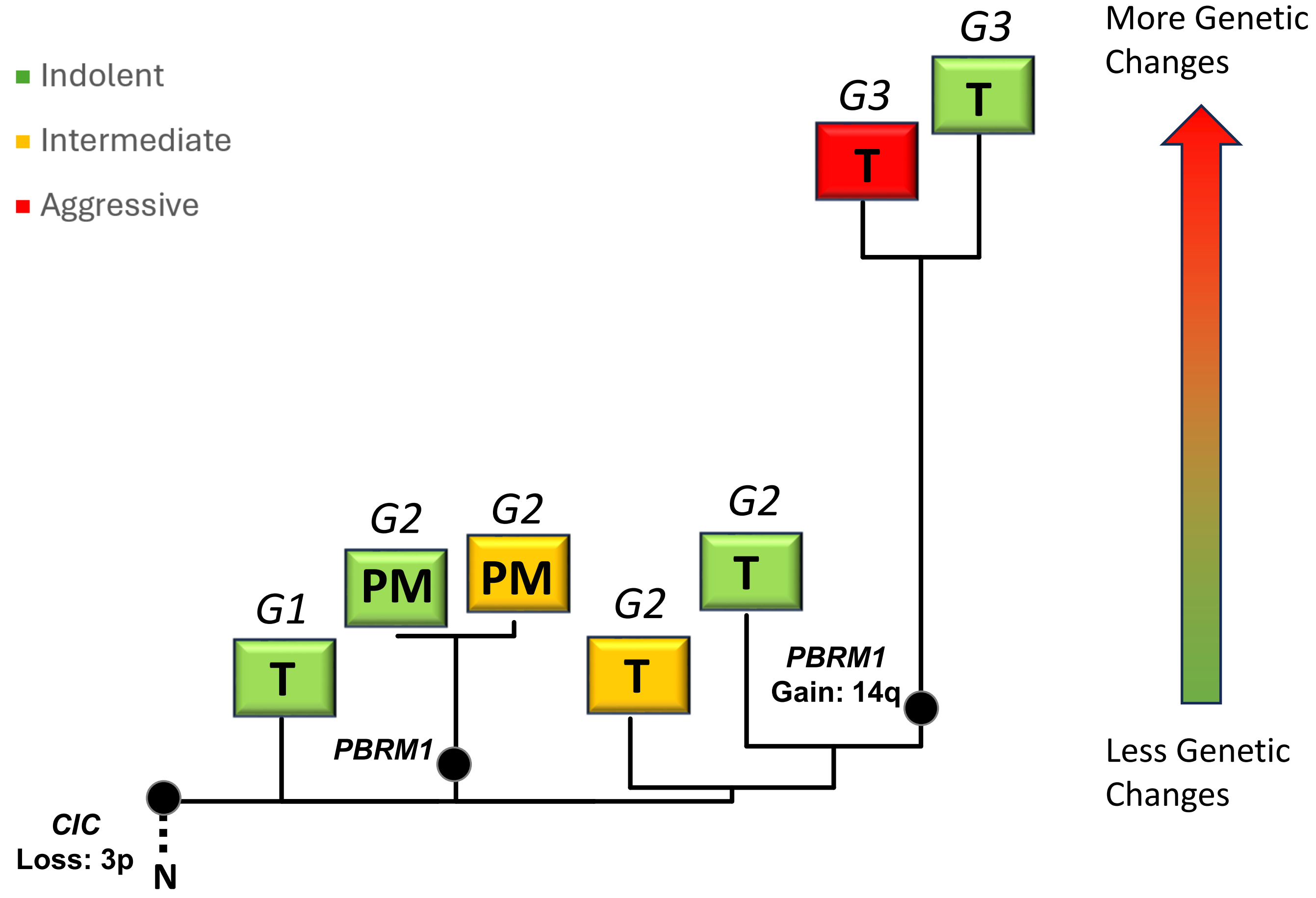

Case 4 (KC01165)

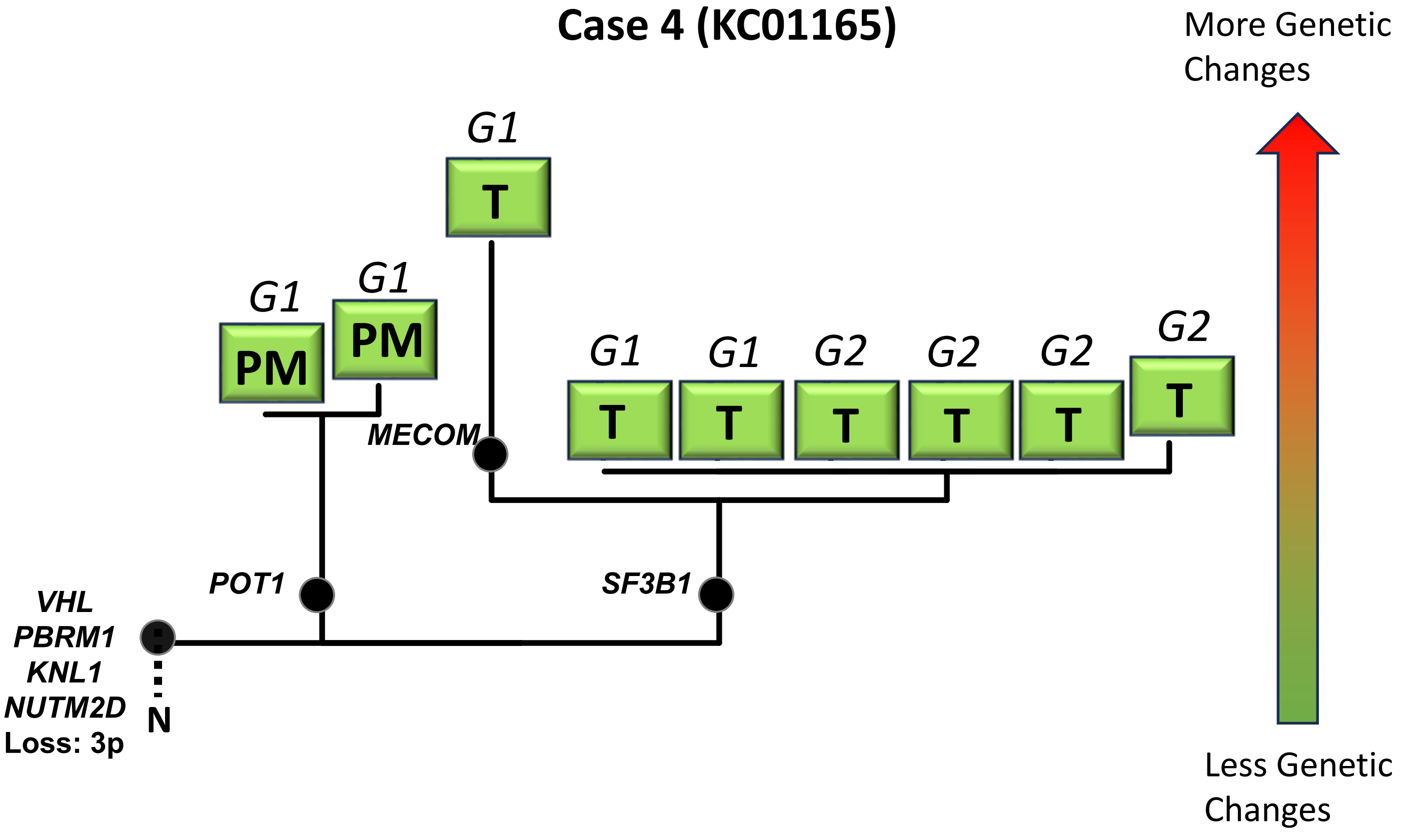

Supplementary Figure legends

Supplementary Figure 1. Central review of histopathological slides of primary tumors. Stacked figure comparing tumor grade assigned to primary tumors based on centralized review of the representative highest-grade slide versus the grade reported on standardized templates by participating institutions, using Fisher's exact test. Among 71 patients with primary tumor tissues, one patient had bilateral kidney tumors, and the assigned grade for that patient corresponds to the highest grade observed. Additionally, two patients did not have a reported grade on their de-identified clinical data sheet and were removed from analyses.

Supplementary Figure 2. Presence of PM is an independent predictor of prolonged survival in multiple variable analyses. Forest plot showing multiple variable Cox proportional hazards analyses between mRCC patients with or without PM. IMDC, International metastatic renal cell carcinoma database consortium; No PM, No pancreatic metastases cohort; PM, pancreatic metastases cohort.

Supplementary Figure 3. PBRM1 status is an independent predictor of prolonged survival in multiple variable analyses. Forest plot showing multiple variable Cox proportional hazards analyses between mRCC patients with PBRM1-deficient or PBRM1+ tumors. Due to potential collinearity concerns between IMDC risk profile and ECOG status, these variables were not included simultaneously in the model. ECOG, Eastern Cooperative Oncology Group; IMDC, International metastatic renal cell carcinoma database consortium; PBRM1-, patients with PBRM1-deficient tumor(s); PBRM1+, patients without evidence of PBRM1 loss.

Supplementary Figure 4. PM Status remains an independent predictor of prolonged survival in patients who develop extrapancreatic metastases. (A) Kaplan-Meier and Cox proportional hazards analyses of OS and (B) multiple variable analyses for mRCC patients with PM who also developed extrapancreatic metastases compared to mRCC patients without PM. Patients with missing survival data were excluded. CI, confidence interval; HR, hazard ratio; IMDC, International metastatic renal cell carcinoma database consortium; No PM, cohort without pancreatic metastases; OS, overall survival; PM, pancreatic metastases cohort.

Supplementary Figure 5. Kaplan-Meier analyses of ccRCC patients who had metastases to pancreas plus additional sites, according to their PBRM1 status. Kaplan-Meier and Cox proportional hazards analyses of (A) OS from time of metastases diagnosis according to PBRM1 status, and (B) time on first line systemic treatment (Angiogenesis inhibitor or IO therapy) for patients with PBRM1-deficient tumors. Patients with missing survival or PBRM1 status data were excluded. CI, confidence interval; HR, hazard ratio; IO, immuneoncology therapy; OS, overall survival; PBRM1-, PBRM1-deficient tumor; PBRM1+, tumor with retained PBRM1; TOT, time-on-treatment.

Supplementary Figure legends

Supplementary Figure 6. Differential mTORC1 activation among PBRM1-mutant metastases according to metastatic site. Boxplot of PS6 H-score in PBRM1-deficient PM compared to PBRM1-deficient non-PM samples. Samples with missing values were excluded. mTORC1, mTOR complex 1; Non-PM, non-pancreatic metastases; PM, pancreatic metastases; PS6, Phospho-S6 ribosomal protein. ***P < 0.001 by using Wilcoxon rank-sum test.

Supplementary Figure 7. Concordance analyses for PBRM1-deficient primary tumors with matched metastases (PM and non-PM). Sunburst charts showing PBRM1 status in (A) PM samples and their matched PBRM1-deficient primary tumors, and (B) Non-PM samples and their matched PBRM1-deficient primary tumors. Concordance between primary and metastatic samples was compared between groups using the Fisher–Freeman–Halton exact test. Non-PM, non-pancreatic metastases; PBRM1-, PBRM1-deficient tumor; PM, pancreatic metastases.

Supplementary Figure 8. Phylogenic trees of PM. Phylogenetic trees for Case 1 and Case 4 (see Figure 9) where branch length is proportional to genetic change. PM samples in each case from the same metastasis. G, grade; N, normal tissue; T, primary tumor; PM, pancreatic metastases.
